# Comparative Genomics of *Streptococcus thermophilus* Support Important Traits Concerning the Evolution, Biology and Technological Properties of the Species

**DOI:** 10.3389/fmicb.2019.02916

**Published:** 2019-12-20

**Authors:** Voula Alexandraki, Maria Kazou, Jochen Blom, Bruno Pot, Konstantinos Papadimitriou, Effie Tsakalidou

**Affiliations:** ^1^Laboratory of Dairy Research, Department of Food Science and Human Nutrition, Agricultural University of Athens, Athens, Greece; ^2^Bioinformatics and Systems Biology, Justus Liebig University Giessen, Giessen, Germany; ^3^Research Group of Industrial Microbiology and Food Biotechnology (IMDO), Department of Bioengineering Sciences (DBIT), Vrije Universiteit Brussel, Brussels, Belgium

**Keywords:** lineage, horizontal gene transfer, genomic islands, milk, yogurt, cheese, pan genome, CRISPR

## Abstract

*Streptococcus thermophilus* is a major starter for the dairy industry with great economic importance. In this study we analyzed 23 fully sequenced genomes of *S. thermophilus* to highlight novel aspects of the evolution, biology and technological properties of this species. Pan/core genome analysis revealed that the species has an important number of conserved genes and that the pan genome is probably going to be closed soon. According to whole genome phylogeny and average nucleotide identity (ANI) analysis, most *S. thermophilus* strains were grouped in two major clusters (i.e., clusters A and B). More specifically, cluster A includes strains with chromosomes above 1.83 Mbp, while cluster B includes chromosomes below this threshold. This observation suggests that strains belonging to the two clusters may be differentiated by gene gain or gene loss events. Furthermore, certain strains of cluster A could be further subdivided in subgroups, i.e., subgroup I (ASCC 1275, DGCC 7710, KLDS SM, MN-BM-A02, and ND07), II (MN-BM-A01 and MN-ZLW-002), III (LMD-9 and SMQ-301), and IV (APC151 and ND03). In cluster B certain strains formed one distinct subgroup, i.e., subgroup I (CNRZ1066, CS8, EPS, and S9). Clusters and subgroups observed for *S. thermophilus* indicate the existence of lineages within the species, an observation which was further supported to a variable degree by the distribution and/or the architecture of several genomic traits. These would include exopolysaccharide (EPS) gene clusters, Clustered Regularly Interspaced Short Palindromic Repeats (CRISPRs)-CRISPR associated (Cas) systems, as well as restriction-modification (R-M) systems and genomic islands (GIs). Of note, the histidine biosynthetic cluster was found present in all cluster A strains (plus strain NCTC12958^T^) but was absent from all strains in cluster B. Other loci related to lactose/galactose catabolism and urea metabolism, aminopeptidases, the majority of amino acid and peptide transporters, as well as amino acid biosynthetic pathways were found to be conserved in all strains suggesting their central role for the species. Our study highlights the necessity of sequencing and analyzing more *S. thermophilus* complete genomes to further elucidate important aspects of strain diversity within this starter culture that may be related to its application in the dairy industry.

## Introduction

Lactic acid bacteria (LAB) include several species, which are extensively used as starters in dairy fermentations (Kongo, 2013). Among them, *Streptococcus thermophilus* constitutes a major starter for the dairy industry. It is primarily used in the production of yogurt, alongside with *Lactobacillus delbrueckii* subsp. *bulgaricus*, but also in the production of several cheese varieties, such as Feta and Mozzarella (Purwandari et al., 2007; Rantsiou et al., 2008; Anbukkarasi et al., 2013). *S. thermophilus* is the only species which was granted the generally recognized as safe (GRAS) status according to the Food and Drug Administration [FDA], 2007 and the qualified presumption of safety (QPS) status according to the European Food Safety Authority [EFSA], 2007 within the *Streptococcus* genus, which consists mainly of commensals and pathogenic species. As it is attested by the large number of pseudogenes identified in the genomes of the *S. thermophilus* strains sequenced so far, the species has undergone significant genome decay probably due to its adaptation to the dairy environment, which is particularly rich in nutrients (Bolotin et al., 2004; Hols et al., 2005; Goh et al., 2011). The regressive evolution of the species has led to genome reduction and simplification of its metabolism (Mayo et al., 2008). The latter is reflected in the deterioration of genes involved, among others, in sugar utilization. *S. thermophilus* has also lost typical streptococcal pathogenic features presumably through strain selection during domestication toward a starter culture (Bolotin et al., 2004; Hols et al., 2005; Goh et al., 2011; Papadimitriou et al., 2015b). Furthermore, the protocooperation with *L. bulgaricus* during the production of yogurt has further shaped the metabolic properties of *S. thermophilus* toward this symbiotic relationship (Mayo et al., 2008).

Typical technological features of *S. thermophilus*, such as milk acidification, lactose and galactose utilization, proteolytic activity and exopolysaccharide (EPS) production, contribute in shaping the organoleptic characteristics of the final products (Cui et al., 2016). In addition, the stress responses of the species define its performance under the unfavorable conditions prevailing during food production (Zotta et al., 2008; Cui et al., 2016). *S. thermophilus* also carries Clustered Regularly Interspaced Short Palindromic Repeats (CRISPRs)-CRISPR associated (Cas) (CRISPR-Cas) and restriction-modification (R-M) systems, which may contribute to competitiveness in microbial food ecosystems and resistance against bacteriophages and other parasitic DNA (Horvath and Barrangou, 2010; Dupuis et al., 2013). Moreover, genes in genomic islands (GIs), which have been acquired most probably through horizontal gene transfer (HGT) events, may ascribe a number of adaptive traits to *S. thermophilus* and could be related to technological characteristics, such as EPS production, bacteriocin biosynthesis, and protocooperation (Liu et al., 2009; Eng et al., 2011).

Another topic that has attracted some attention concerning *S. thermophilus* was the biodiversity of strains within the species. Original studies used typing techniques like random amplification of polymorphic DNA-PCR (RAPD-PCR) or pulsed-field gel electrophoresis (PFGE), while more recent ones used multilocus sequence typing (MLST) (Moschetti et al., 1998; Giraffa et al., 2001; Mora et al., 2002; Ercolini et al., 2005; Delorme et al., 2010, 2017; Yu et al., 2015). Application of MLST in *S. thermophilus* had to be optimized to increase discriminating power, given the fact that the species may exhibit limited genetic variability (Delorme et al., 2017). In this study the authors reported 116 sequence types and the existence of groups of strains based on phylogenetic analysis of concatenated sequences of housekeeping genes. Additional analysis revealed clustering of strains based on core genome and CRISPR spacer analysis of 25 sequenced strains (both complete and partial). The authors reported that the clustering based on MLST and whole genome analysis was in agreement but differed from that of CRISPR analysis. With the MLST scheme developed and the wide sample of *S. thermophilus* strains (*n* = 178), it was feasible to detect relationship between strains and geographic location.

Furthermore, due to the economic importance of *S. thermophilus* as a starter, a number of groundbreaking studies have been conducted in an attempt to elucidate the genetic basis behind the physiological and the metabolic properties of the species, which define its technological and probiotic potential. Comparative genomics of *S. thermophilus* was carried out early on and provided significant information about its adaptation to the milk environment and technological traits (Bolotin et al., 2004; Hols et al., 2005; Goh et al., 2011). However, these studies relied only on a limited number of genome sequences. The current accumulation of completely sequenced *S. thermophilus* genomes can increase the predictive power of comparative analysis and enhance the interpretation of the acquired data about the genome architecture, functionality and evolution. Furthermore, the advancement of bioinformatics tools and the demand of the dairy industry for novel starter strains render an updated analysis of the species essential. In the present study, the results of an in depth analysis of 23 complete *S. thermophil*us genomes are presented, focusing on main technological features of the species.

## Materials and Methods

### Strains

The 23 *S. thermophilus* genomes designated as “complete” up to RefSeq release 88, were selected for analysis in this study (Table 1). The majority of *S. thermophilus* strains have been isolated from yogurt (strains LMG 18311, CNRZ1066, LMD-9, MN-ZLW-002, MN-BM-A01, KLDS SM, KLDS 3.1003, and ACA-DC 2) and milk (strains JIM 8232, SMQ-301, ND03, ND07, B59671, EPS, GABA, and NCTC12958^T^). Furthermore, three isolates, namely strains S9, MN-BM-A02, and CS8, derived from traditional Chinese dairy products. More specifically, MN-BM-A02 was isolated from Fan, a traditional Chinese cheese-like product, while CS8 from Rubing, a Chinese fresh goat milk cheese. Finally, strains APC151 and ST3 were isolated from fish intestine and commercial dietary supplements, respectively.

**TABLE 1:** *Streptococcus thermophilus* strains with complete genomes analyzed in this study.

**Strain**	**GenBank accession**	**Isolation source**	**Sequencing technology**	**References**
LMG 18311	NC_006448	Commercial yogurt	Random shotgun sequencing	Bolotin et al., 2004
CNRZ1066	NC_006449	Commercial Yogurt	Random shotgun sequencing	Bolotin et al., 2004
LMD-9	NC_008532	Yogurt	Whole-genome shotgun sequencing	Makarova et al., 2006
ND03	NC_017563	Naturally fermented yak milk	454; Solexa	Sun et al., 2011
JIM 8232	NC_017581	Raw milk	SOLiD; Sanger	Delorme et al., 2011
MN-ZLW-002	NC_017927	Traditional yogurt block	454; Solexa	Kang et al., 2012
ASCC 1275	NZ_CP006819	–	454	Wu et al., 2014
SMQ-301	NZ_CP011217	Milk	Illumina; PacBio	Labrie et al., 2015
MN-BM-A02	NZ_CP010999	Dairy fan	454 GS FLX	Shi et al., 2015
MN-BM-A01	NZ_CP012588	Traditional yogurt block	PacBio RS	Bai et al., 2016
KLDS 3.1003	NZ_CP016877	Traditional yogurt	Illumina	Evivie et al., 2017
ACA-DC 2	NZ_LT604076	Traditional yogurt	Illumina HiSeq2500; PacBio RSII	Alexandraki et al., 2017
APC151	NZ_CP019935	Fish intestine	PacBio RS	Linares et al., 2016, 2017
B59671	NZ_CP022547	Raw milk	PacBio RS	Renye et al., 2017
KLDS SM	NZ_CP016026	Traditional yogurt	Illumina	Li et al., 2018
DGCC 7710	NZ_CP025216	Dairy culture	Illumina MiSeq; PacBio RS	Hatmaker et al., 2018
S9	NZ_CP013939	Traditional dairy	PacBio	–
CS8	NZ_CP016439	Rubing	PacBio	–
ND07	NZ_CP016394	Naturally fermented yak milk	PacBio RSII	–
EPS	NZ_CP025400	Milk	PacBio RS	–
GABA	NZ_CP025399	Milk	PacBio RS	–
ST3	NZ_CP017064	Commercial dietary supplements	PacBio RS	–
NCTC12958^T^	NZ_LS483339	Milk	–	–

### Comparative and Evolutionary Genomics

ProgressiveMauve was used for the whole genome alignment of the 23 *S. thermophilus* strains analyzed in this study (Darling et al., 2010). GenSkew online application was employed in the evaluation of the chromosomal inversions in strains EPS, MN-BM-A01, and MN-ZLW-002^1^. The pan/core genome analysis was performed with the bacterial pan genome analysis (BPGA) pipeline v.1.3 using USEARCH v.9.2.64 for clustering gene families (Edgar, 2010) with a 60% sequence identity cut-off and 20 random permutations of genomes to avoid any bias in the sequential addition of new genomes. The protein coding sequences assigned in the core, accessory and unique gene families were further analyzed for clusters of orthologous groups (COG) categories within the BPGA pipeline (Chaudhari et al., 2016). Alternatively, protein coding sequences of *S. thermophilus* strains were also analyzed for COG categories with the eggnog-mapper based on eggNOG v.4.5 orthology database, as highlighted in the text (Huerta-Cepas et al., 2016, 2017). The EDGAR tool was also employed to assist analysis of orthologs whenever necessary, as well as for core genome phylogenetic analysis among *S. thermophilus* strains (Blom et al., 2016). For the latter, the alignments of the core gene sets were executed with MUSCLE and concatenated to one complete core alignment, which was used to generate the phylogenetic tree by the neighbor-joining method as implemented in the PHYLIP package. The consensus tree topology was verified by 100 bootstrap iterations. The EDGAR software was also exploited for the investigation of the relatedness among *S. thermophilus* strains through the construction of average nucleotide identity (ANI) heat map. The ANI values were computed as described by Goris et al. (2007) and as implemented in the JSpecies package (Richter and Rossello-Mora, 2009). The resulting phylogenetic distance values were arranged in an ANI matrix, clustered according to their distance patterns and visualized as a color-coded heatmap, with dark and light orange for high and low similarity regions, respectively. Box Plot Generator was employed for the visualization of genome size differences between the two clusters of the *S. thermophilus* strains^2^. Statistical differences in genome size were accessed with the Mann–Whitney *U* Test for *p* < 0.05. The quality of the genome assemblies was evaluated with the microbial genomes atlas (MiGA) webserver (Rodriguez-R et al., 2018). The COG frequency and the accessory genes presence/absence heatmaps were generated with the RStudio using the heatmap.2 function included in the Gplots package^3^. Kyoto encyclopedia of genes and genomes (KEGG) orthology and links Annotation (KOALA) was employed for K number assignment to *S. thermophilus* protein coding sequences (Kanehisa et al., 2016b), while KEGG Mapper tools were exploited for further processing of KO annotations (Kanehisa et al., 2016a). The PHASTER web server was used for the identification of putative prophages (Arndt et al., 2016). The comparison of the EPS gene clusters was performed with the Easyfig tool (Sullivan et al., 2011). The transporters were determined using the TransportDB database (Elbourne et al., 2017). The CRISPRs were identified with CRISPRFinder web tool (Grissa et al., 2007), while comparison of the predicted spacers was performed with CD-HIT Suite (Huang et al., 2010). The REBASE database was used for verifying the R-M systems (Roberts et al., 2015). Finally, the GIs were obtained through the IslandViewer 4 web-based resource (Bertelli et al., 2017). For our analysis, GIs characterized as integrated by the IslandViewer tool were analyzed.

## Results and Discussion

### General Genomic Features

The general genome features of the 23 *S. thermophilus* strains used in this study are presented in Table 2. The chromosome length of the strains ranges between 1.73 and 2.10 Mbp, with an average of 1.85 Mbp, while the % GC content is around 39.0. The number of genes varied between 1,847 and 2,237 including protein coding sequences that varied between 1,555 and 1,854. The percentage of pseudogenes ranged between 9.64 and 13.97%. These variations in genome size, gene and pseudogene content indicate important differences in both gene gain and gene loss events during the evolution of the different strains. It has been previously reported that *S. thermophilus* owns some of the smallest genomes within streptococci while *Streptococcus salivarius* some of the largest (Delorme et al., 2015). Based on the complete genome sequences within the salivarius group we found that the percentage of pseudogenes of *S. salivarius* (12 complete genomes) may reach up to 4% while the percentage of pseudogenes of *Streptococcus vestibularis* NCTC12167, the only strain with a complete genome, was around 8%. These findings suggest a variable degree of evolution through genome decay within the group. Beyond the salivarius group, high percentages of pseudogenes have also been reported for *Streptococcus macedonicus* and *Streptococcus infantarius* that are also associated with the dairy environment (Jans et al., 2013a; Papadimitriou et al., 2014). A high number of pseudogenes has also been reported for certain strains of *Streptococcus pneumoniae* (see for example the studies by Junges et al., 2019; Scott et al., 2019). Interestingly, extensive genome decay seems to be compatible with adaptation in milk (Bolotin et al., 2004; Hols et al., 2005; Jans et al., 2013a; Papadimitriou et al., 2014) or a pathogenic lifestyle (Lerat and Ochman, 2005). Obviously more research is needed to appreciate the strains/species within streptococci that have evolved through reductive processes and to test whether this evolution path can be correlated with the niches they occupy.

**TABLE 2:** General genome features of *S. thermophilus* strains with complete genomes analyzed in this study.

**Strain**	**Genome size (bp)**	**GC (%)**	**Genes**	**Proteins**	**rRNA**	**tRNA**	**Pseudogenes (% of pseudogenes)**	**Predicted essential genes^1^**	**Genome completeness (%)^2^**	**Corrected genome completeness (%)^3^**
NCTC12958^T^	2,102,271	39.0	2,237	1,854	15	56	308 (13.77)	106	95.5	100.0
JIM 8232	1,929,905	38.9	2,033	1,748	18	67	196 (9.64)	104	93.7	98.1
KLDS 3.1003	1,899,956	38.9	2,037	1,676	18	68	271 (13.30)	105	94.6	99.1
MN-BM-A01	1,876,516	39.1	2,023	1,661	18	67	273 (13.49)	104	93.7	98.1
ND07	1,869,510	39.0	1,996	1,684	15	57	236 (11.82)	105	94.6	99.1
ST3	1,865,056	39.0	1,982	1,638	18	69	253 (12.76)	106	95.5	100.0
SMQ-301	1,861,792	39.1	1,993	1,684	18	67	220 (11.04)	106	95.5	100.0
GABA	1,857,468	39.1	1,952	1,621	18	68	241 (12.35)	106	95.5	100.0
KLDS SM	1,856,787	39.1	1,984	1,671	18	67	224 (11.29)	106	95.5	100.0
LMD-9	1,856,368	39.1	1,993	1,674	18	67	230 (11.54)	105	94.6	99.1
DGCC 7710	1,851,207	39.0	1,962	1,657	15	56	230 (11.72)	106	95.5	100.0
MN-BM-A02	1,850,434	39.0	1,977	1,677	15	57	224 (11.33)	106	95.5	100.0
MN-ZLW-002	1,848,520	39.1	1,982	1,695	15	57	211 (10.65)	105	94.6	99.1
ASCC 1275	1,845,495	39.1	1,974	1,666	15	55	234 (11.85)	106	95.5	100.0
APC151	1,839,134	39.1	1,982	1,687	18	67	206 (10.39)	106	95.5	100.0
ND03	1,831,949	39.0	1,968	1,692	15	57	200 (10.16)	105	94.6	99.1
B59671	1,821,173	39.1	1,925	1,567	18	67	269 (13.97)	106	95.5	100.0
EPS	1,812,305	39.0	1,937	1,608	18	67	240 (12.39)	106	95.5	100.0
LMG 18311	1,796,846	39.1	1,925	1,621	18	67	215 (11.17)	105	94.6	99.1
CNRZ1066	1,796,226	39.1	1,936	1,638	18	67	209 (10.80)	106	95.5	100.0
CS8	1,791,656	39.0	1,924	1,641	15	57	207 (10.76)	106	95.5	100.0
S9	1,787,436	39.1	1,922	1,630	18	67	203 (10.56)	106	95.5	100.0
ACA-DC 2	1,731,838	39.2	1,847	1,555	15	56	217 (11.75)	106	95.5	100.0

*Cluster A strains start with strain JIM 8232 and end with strain ND03. Cluster B strains start with strain B59671 and end with strain ACA-DC 2. ^1^Out of 111 essential genes. ^2^Genome completeness as calculated by MiGA webserver considering 111 essential genes. ^3^Corrected genome completeness considering 106 essential genes after the omission of *glyS*, *proS*, *pheT*, *nahD*, *rpoC1* missing from all *S. thermophilus* genomes from the list of essential genes used by MiGA webserver.*

Fourteen out of 23 strains carry 18 rRNA genes and the rest carry 15. Interestingly, strains with 18 rRNA genes also own a higher number of tRNA genes (ranging from 67 to 69) compared to strains with 15 rRNAs which own fewer tRNA genes (ranging from 55 to 57). A general comment that can be made about this difference is that strains with a higher number of rRNA and tRNA genes could potentially exhibit a higher growth/metabolic rate (Wassenaar and Lukjancenko, 2014).

Comparison of the chromosomal architecture of the 23 *S. thermophilus* strains was performed through full-length sequence alignments (Supplementary Figure S1). All strains were synchronized from the *dnaA* so as to simplify the alignment. Analysis revealed a high degree of conservation among different strains. However, strain-specific differences could also be detected. More specifically, low similarity regions, represented as white regions inside the local collinear blocks (LCBs), were found in all strains. Furthermore, many unique regions, represented as blank spaces between the LCBs, were also identified in all strains. In strain EPS a large inversion (1.47 Mbp) was present, while in strains MN-BM-A01 and MN-ZLW-002, a ∼300 kbp inverted region was identified between coordinates 768,310–1,068,868 and 740,416–1,040,999 bp, respectively. These inversions could be either genuine or could be ascribed to assembly artifacts. If the first is true, our observations may correspond to an inversion around the origin of replication for strain EPS, or to an inversion around the terminus of replication for strains MN-BM-A01 and MN-ZLW-002. Such inversions have been described before for bacterial genomes as part of their evolution (Eisen et al., 2000; Darling et al., 2008; Repar and Warnecke, 2017).

### Pan/Core Genome Analysis and Phylogenomics

**FIGURE 1:**
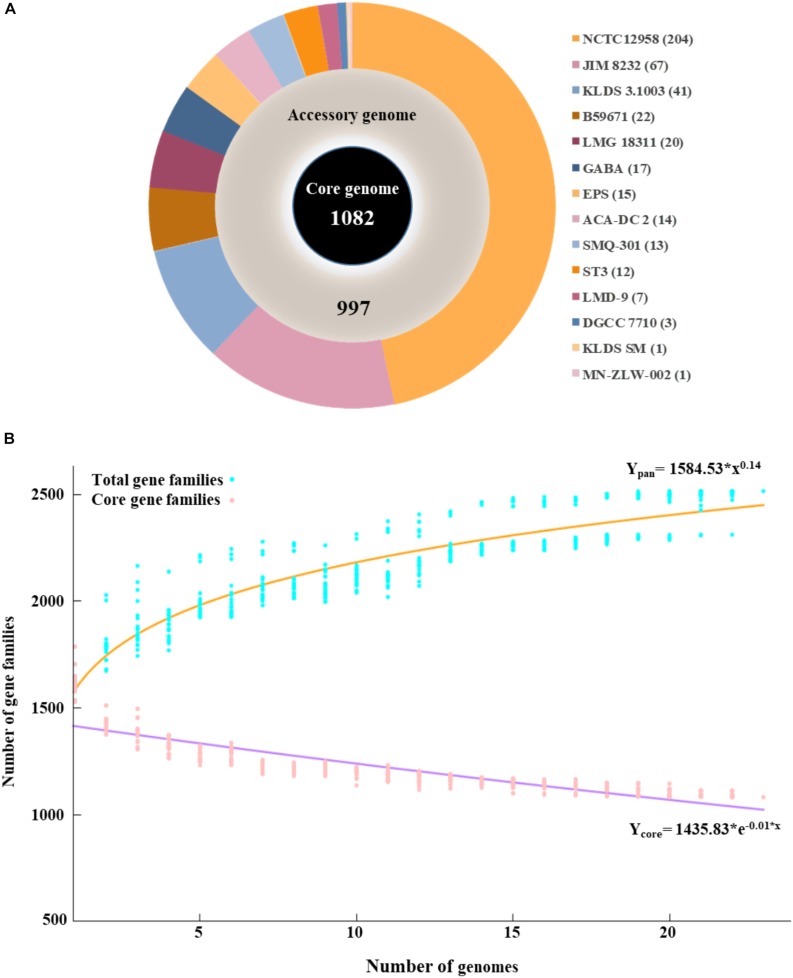
Graphical presentation of core genome (inner black circle), accessory genome (middle gray circle) and unique genes (outer multicolored circle) of the 23 *S. thermophilus* strains. The number of unique genes for each strain is presented in the parentheses **(A)**. Pan and core genome plots of *S. thermophilus* strains analyzed **(B)**.

The pan genome of the 23 *S. thermophilus* strains contains a total number of 2,516 genes, including 1,082 and 997 genes in the core and accessory genomes, respectively (Figure 1A). The number of genes in the accessory genome of each strain varied between 432 and 568 and a total of 437 unique genes (singletons) were identified in 14 strains (Supplementary Table S1 and Figure 1A). According to BPGA analysis, the *b* value of 0.14 in the power-law regression model is indicative of an open pan genome for *S. thermophilus* that is probably going to be closed soon (Figure 1B). This may also be supported by the fact that within the total of unique genes identified in *S. thermophilus* strains, 71% belong to three strains, namely KLDS 3.1003 (*n* = 41), JIM 8232 (*n* = 67), and NCTC12958^T^ (*n* = 204), while strains APC151, ASCC 1275, CNRZ1066, CS8, MN-BM-A01, MN-BM-A02, ND03, ND07, and S9 have no unique genes (Supplementary Tables S1, S2). BPGA analysis also revealed the number of exclusively absent genes per strain (Supplementary Table S1). Core, accessory and unique genes were further classified into COG categories, as implemented within the BPGA pipeline (Supplementary Figure S2). The analysis revealed that approximately 90% of the core, 60% of the accessory and 40% of the unique genes were assigned to various COG categories, with the rest having no prediction. We then excluded the poorly characterized categories R and S from further analysis. The majority of core genes encode proteins involved primarily in housekeeping and metabolic processes. The three most abundant COG categories were J (translation, ribosomal structure, and biogenesis, 12.7%), E (amino acid transport and metabolism, 11.8%), and L (replication, recombination, and repair, 7.3%). In the case of the accessory and unique genes the categories with the highest percentages included categories E, L, and K (transcription) and L, K, and V (defense mechanisms), respectively. In general, accessory and unique genes encoded among others transposases, Cas proteins, R-M systems, glycosyltranferases, polysaccharide biosynthesis proteins, amino acid biosynthesis proteins, proteolytic enzymes, stress related proteins, as well as transporters which may contribute to strain-specific technological traits (please see below).

**FIGURE 2:**
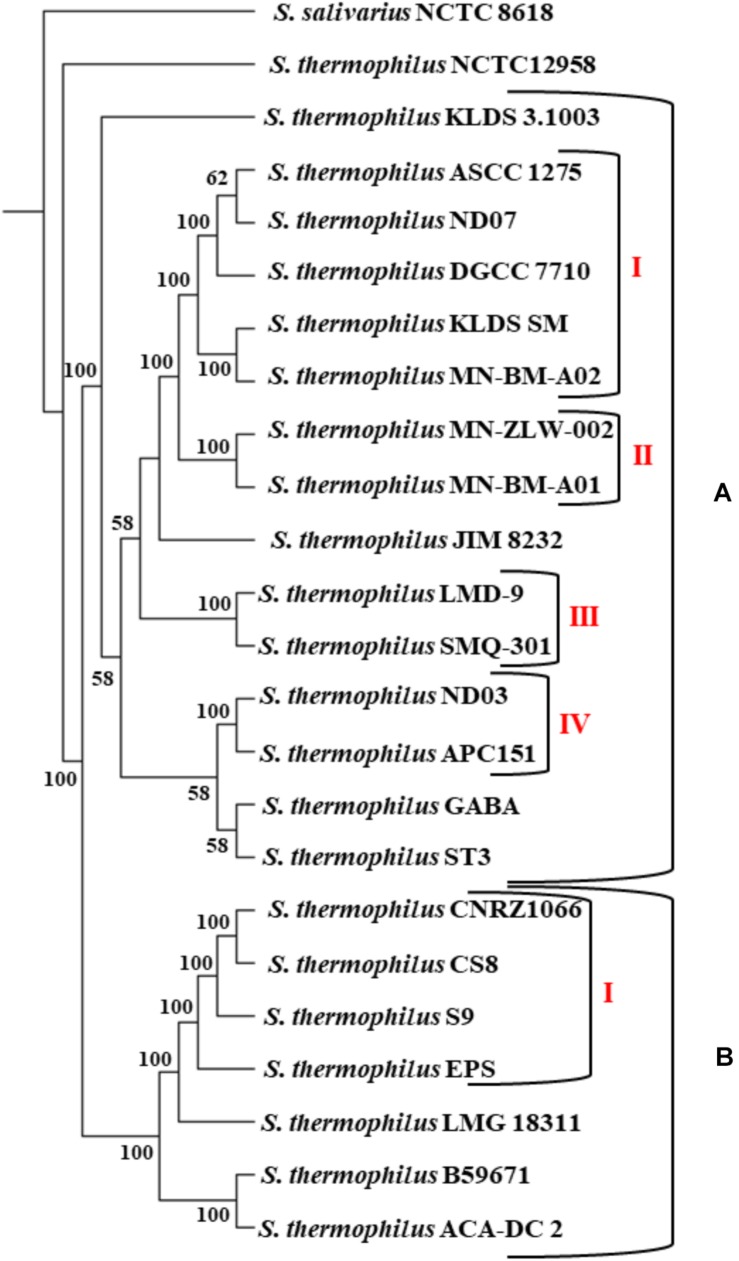
Core genome phylogenetic tree of the 23 *S. thermophilus* strains. Strains were grouped in two clusters **(A,B)**. Subgroups within the clusters are also highlighted (AI, AII, AIII, AIV, and BI). *S. salivarius* NCTC 8618 was used as an outgroup. For branches with less than 50% bootstrap support the bootstrap values are not shown.

**FIGURE 3:**
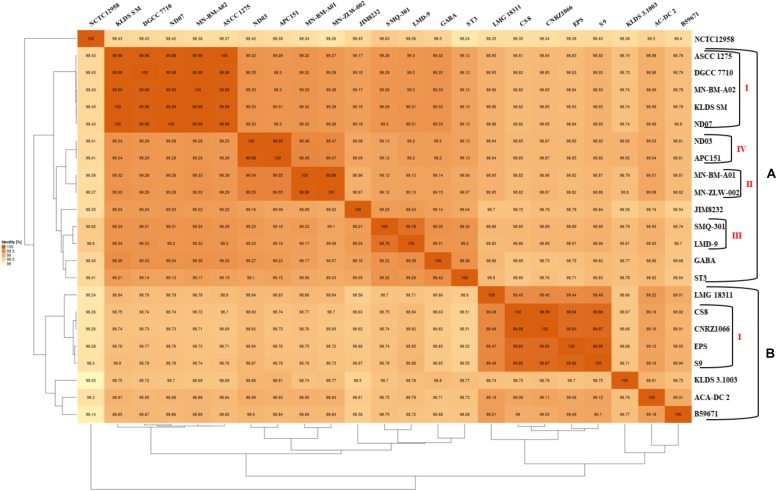
Average nucleotide identity (ANI) phylogenetic tree and heat map visualization of the 23 *S. thermophilus* strains.

The phylogenetic relationship among the *S. thermophilus* strains was determined based on the core genome of the strains and revealed two main clusters containing 15 (APC151, ASCC 1275, DGCC 7710, GABA, JIM 8232, KLDS 3.1003, KLDS SM, LMD-9, MN-BM-A01, MN-BM-A02, MN-ZLW-002, ND03, ND07, SMQ-301, and ST3; cluster A) and seven (ACA-DC 2, B59671, CNRZ1066, CS8, EPS, LMG 18311, and S9; cluster B) strains, respectively, while strain NCTC12958^T^ was placed separately (Figure 2). Moreover, the ANI phylogenetic tree had practically an identical topology to that of the phylogenetic tree (Figure 3). There was only one exception with strain KLDS 3.1003 being placed in cluster B. A more detailed inspection of the potential differences between strains in the two clusters revealed that cluster A strains had larger genomes beyond 1.83 Mbp, while those in cluster B had smaller genomes (Table 2 and Supplementary Figure S3). This difference was found to be statistically significant (*p* < 0.05) suggesting that strains in the two clusters may have been separated by distinct gene gain and/or gene loss events. Within these two main clusters, subgroups of *S. thermophilus* strains could also be identified during both phylogenetic and ANI analysis. These subgroups include strains ASCC 1275, DGCC 7710, KLDS SM, MN-BM-A02, and ND07 (subgroup AI), MN-BM-A01 and MN-ZLW-002 (subgroup AII), LMD-9 and SMQ-301 (subgroup AIII), APC151 and ND03 (subgroup AIV), and finally CNRZ1066, CS8, EPS, and S9 (subgroup BI) (Figures 2, 3). As already mentioned, core genome phylogeny was also previously performed in a dataset of 25 *S. thermophilus* strains employing genomes sequenced to a variable degree of completeness (Delorme et al., 2017). In this study 1,311 core proteins were reported. Of note, an earliest study was performed based on three *S. thermophilus* genome sequences reporting 1,487 core genes (Lefébure and Stanhope, 2007). Our core genome was estimated to consist of 1,082 core proteins. This may suggest a more stringent selection of core proteins during our analysis. Despite the fact that several different strains were analyzed in our study and the study by Delorme et al. (2017), phylogenetic clustering of strains exhibited similarities supporting more or less the distinction we propose between cluster A and B strains and the subgroups observed within them. Differences in the topology of the two phylogenetic trees can be attributed to the different dataset of genomes analyzed as well as the different methods employed to construct the trees. The fact that we concentrated our analysis solely on strains with complete genome sequences presents an important advantage, since we were able to support clustering of strains based on the comparative genomic analysis of additional genomic traits as follows. Completeness of genome sequence is of utmost importance when the presence/absence of specific loci or their exact organization are the main factors for strain diversification.

The subgroups mentioned above appeared at high ANI values (>99.9%) which may suggest relatively subtle genomic differences. Such differences may indicate that strains of the same subgroup may be very similar but may deviate from the strict definition of clones. However, clonal relationships may be masked among strains due to aberrations in genome assembly that may come into play at such high ANI values (Burall et al., 2016). To avoid this pitfall, we investigated the quality of the assemblies of all *S. thermophilus* genomes analyzed in this study using the MiGA webserver (Table 2). Our analysis indicated that from the list of the 111 essential genes used to access genome completeness by MiGA, five (i.e., *glyS*, *proS*, *pheT*, *nhaD*, and *rpoC1*) were systematically missing from all *S. thermophilus* genomes. This observation suggested that they do not belong to the gene pool of the species, which is also supported by data presented previously for essential genes in Firmicutes (Albertsen et al., 2013). We thus corrected the completeness score of the genomes by calculating a total of 106 essential genes. Fifteen genomes received 100% genome completeness. Five genomes missed only *secE*, two missed *secE* plus an additional gene (*rpiX* or *uvrb*) and one missed only *ychF* receiving scores above 98.1%. The presence/absence frequency of *secE* may indicate that it is an accessory gene for *S. thermophilus*. In all cases the completeness scores of *S. thermophilus* genomes suggest perfect or nearly perfect assemblies. This is also corroborated by the quality scores for the genome assemblies that were all found “excellent” by MiGA webserver.

**FIGURE 4:**
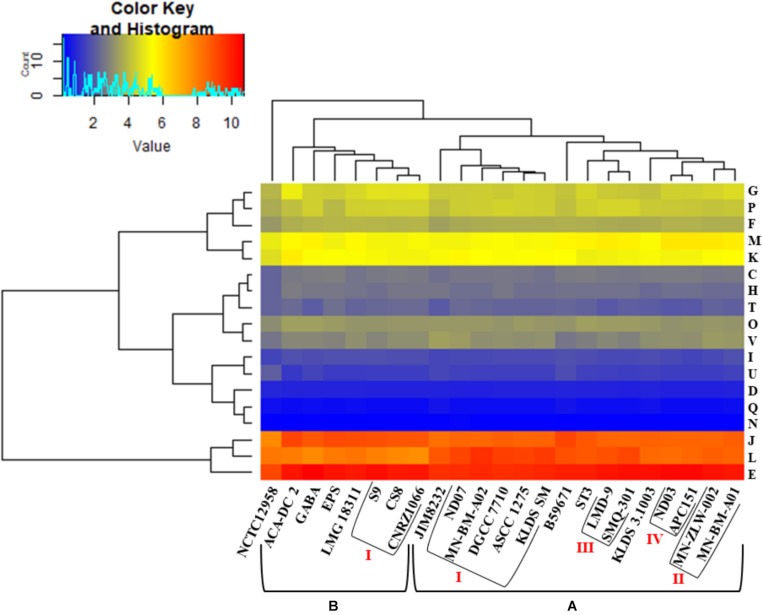
Clusters of orthologous groups (COG) frequency heat map based on a two-dimensional hierarchical clustering. The horizontal axis corresponds to the percentage frequency of proteins involved in the respective COG functional categories: Information storage and processing: translation, ribosomal structure, and biogenesis (J), transcription (K), replication, recombination, and repair (L); cellular processes and signaling: cell cycle control, cell division, chromosome partitioning (D), cell wall/membrane/envelope biogenesis (M), cell motility (N), post-translational modification, protein turnover, chaperones (O), signal transduction mechanisms (T), intracellular trafficking, secretion, and vesicular transport (U), defense mechanisms (V); metabolism: energy production and conversion (C), amino acid transport and metabolism (E), nucleotide transport and metabolism (F), carbohydrate transport and metabolism (G), coenzyme transport and metabolism (H), lipid transport and metabolism (I), inorganic ion transport and metabolism (P), secondary metabolites biosynthesis, transport and catabolism (Q). The vertical axis shows the 23 *S. thermophilus* strains. Strains were grouped in two clusters **(A,B)**. Subgroups within the clusters are also highlighted (AI, AII, AIII, AIV, and BI). Categories R and S, concerning poorly characterized proteins, were not included in the analysis.

Hierarchical clustering of the COG frequency heat map generated for all *S. thermophilus* strains also supported the existence of the clusters and subgroups mentioned above, with minor alterations (Figure 4). Strains GABA and B59671 were placed in opposite clusters, while strains of the BI subgroup were associated more loosely (i.e., not forming a distinct subgroup). The most abundant category in all strains was E, followed by J and L. The prevalence of the E category may support adaptation of *S. thermophilus* to milk and the necessity of the organism to use amino acids from the environment.

**FIGURE 5:**
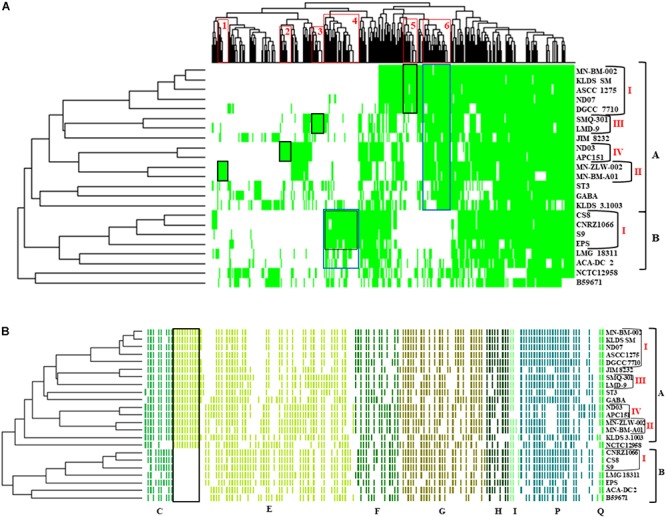
Presence/absence heat map and two-dimensional hierarchical clustering of the 23 *S. thermophilus* strains accessory genome **(A)**. Colored areas represent the presence of genes in the respective *S. thermophilus* strains, while white areas indicate the absence of genes. Accessory genes clustered according to their presence in specific subgroups or clusters of strains (gene clusters 1 to 6) are highlighted with red frames on the *x* axis. The presence of a specific gene cluster in cluster A or B as well as specific subgroups of strains is highlighted with blue or black frames, respectively. Presence/absence heat map and hierarchical clustering of *S. thermophilus* strains based on accessory genes with clusters of orthologous groups (COG) assignment involved in metabolism (categories C, E, F, G, H, I, P, and Q) **(B)**. Colored areas represent the presence of genes in the respective *S. thermophilus* strains, while white areas indicate the absence of genes. Genes implicated in the biosynthesis of histidine are highlighted with a black frame. Of note, panel **(B)** is a composite figure of an excel generated table manually colored, while clustering was exported from RStudio. This was necessary to achieve clustering of genes grouped based on COG categories.

The presence/absence heat map of the accessory genes of *S. thermophilus* strains supported once again the existence of clusters A and B (Figure 5A). The analysis allowed the identification of genes, which may contribute to the grouping of the strains. As shown in the horizontal axis of the heat map, genes within clusters 4 and 6 are characteristic of clusters B and A, respectively. Moreover, genes of clusters 1, 2, 3, 5, and 4 seem to be present in specific subgroups, namely AII, AIV, AIII, AI, and BI, respectively. Further analysis of the accessory proteins, specifically of those involved in metabolic processes, revealed that cluster A strains (including NCTC12958^T^) carry the entire set of genes responsible for the biosynthesis of histidine that are basically absent from cluster B (Figure 5B). Based on these findings it is plausible to state that strains of *S. thermophilus* exhibit lineage-type relationships.

### Lactose and Galactose Metabolism

*Streptococcus thermophilus* ferments preferentially lactose over glucose (Geertsma et al., 2005). Lactose is the main carbohydrate of milk and therefore constitutes the primary carbon and energy source for *S. thermophilus*, due to the adaptation of the microorganism to this particular niche (Bolotin et al., 2004; Hols et al., 2005; Goh et al., 2011). The genes implicated in the fermentation of lactose and galactose are organized in two adjacent operons (*galRKTEM-lacSZ*) (Vaughan et al., 2001). We found the complete locus in all *S. thermophilu*s strains analyzed, with the exception of three strains in which *lacS* (strains B59671 and KLDS 3.1003) or *galR* (strain NCTC12958^T^) are putative pseudogenes (Supplementary Table S3). The importance of these inactivations needs to be experimentally investigated, but the high degree of conservation of the *gal-lac* gene clusters among the different *S. thermophilus* strains, both at sequence and organization levels, reveals its importance in the catabolism of lactose in milk. Apart from *galE* coding for the enzyme UDP-glucose 4-epimerase that is located in the Leloir gene cluster, a second or even a third distal *galE* gene was identified in certain strains (Supplementary Table S3). It has been demonstrated that the activity of this enzyme is positively correlated with the biosynthesis of precursors for EPS production in EPS producing Gal^–^ *S. thermophilus* strains (Degeest and De Vuyst, 2000). Furthermore, the galactose moiety generated by the hydrolysis of lactose is translocated outside the cell via the dedicated antiporter LacS, which is implicated in the uptake of lactose in exchange to galactose (Vaughan et al., 2003). The majority of *S. thermophilus* strains are unable to metabolize both free and intracellularly produced galactose, probably either due to insufficient activities of *galK* and *galM* genes or due to mutations in the *galR-galK* promoter region, which may interfere with the expression levels of the respective enzymes (De Vin et al., 2005; Vaillancourt et al., 2008; Anbukkarasi et al., 2014; Sørensen et al., 2016). Recently, Xiong et al. (2019b) demonstrated that the Gal^+^ phenotype of *S. thermophilus* depends upon the expression of the *gal* operon, which can be widely affected by a single point mutation at the -9 box in the *galK* promoter. Since the accumulation of galactose in the medium by *S. thermophilus* may be important from a technological or nutritional perspective (Giaretta et al., 2018), we examined the presence of the mutation at the -9 box in the *galK* promoter in the strains analyzed. Accordingly, only B59671, CS8, EPS, and NCTC12958^T^ seem to be able to catabolize galactose, as they own the relevant G to A mutation in the position -9 of the -10 box related Gal^+^ phenotype (data not shown). However, experimental verification is required to validate this prediction.

### Biosynthesis of EPS

**FIGURE 6:**
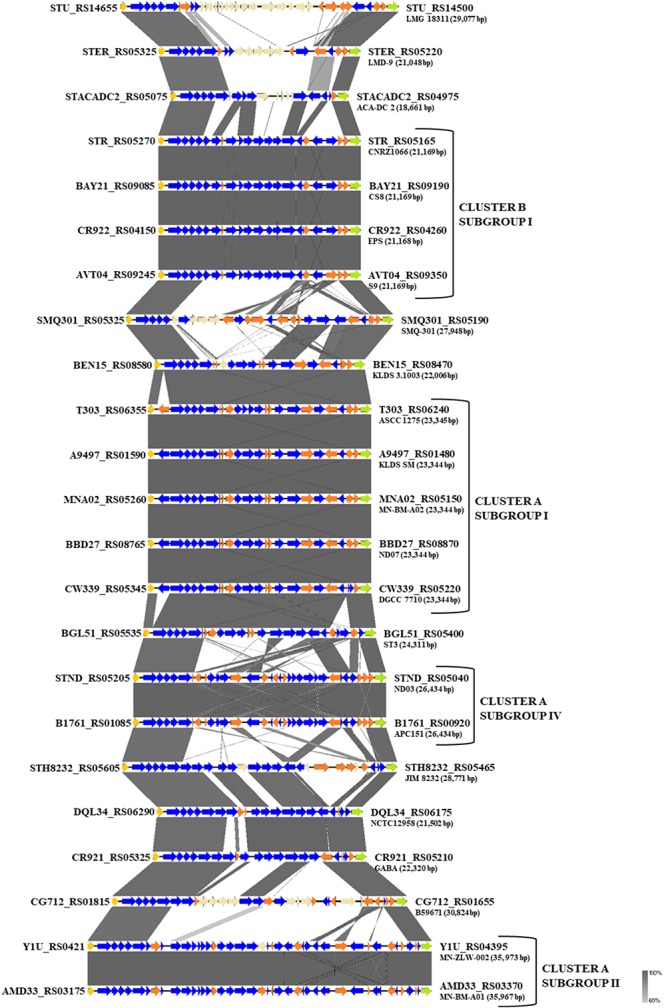
Multiple sequence alignment of the exopolysaccharide (EPS) gene clusters of the 23 *S. thermophilus* strains after BLASTN analysis. Gray shading represents the % identity among the nucleotide sequences according to the color gradient presented at the lower right corner of the figure. Protein coding genes are highlighted in dark blue, putative pseudogenes in orange, the *deoD* in yellow, the transporter gene in green and the unique genes for each *S. thermophilus* strain in beize. Clusters and subgroups of strains are highlighted.

One of the key technological properties of *S. thermophilus* is the production of EPS, which has been related to desirable textural properties and reduced syneresis in fermented dairy products (Lluis-Arroyo et al., 2014; Han et al., 2016). In a recent study, the EPS clusters of several strains were compared suggesting variations in the gene content of these loci (Cui et al., 2017). Our analysis revealed the presence of EPS gene clusters in all *S. thermophilus* strains examined. The size of the clusters ranged between 18,661 and 35,973 bp and the % GC content (34.3–36.4%) was found to be lower than the % GC content calculated for the complete genomes of all strains (Supplementary Table S4). All clusters are flanked by a purine-nucleoside phosphorylase (*deo*D) and a transporter protein as their boundaries (Figure 6). The alignment of the EPS loci showed that they are highly conserved at the 5′ and the 3′ ends and their differences are located mainly in the middle of the clusters. At the 5′ end, genes *epsA*, *epsB*, *epsC*, and *epsD* were found in all EPS gene clusters and their role has been associated with the regulation of *eps* genes and chain elongation of the EPS molecules (Cui et al., 2017). The adjacent *epsE* gene coding a galactosyl-1-phosphate transferase was found in five out of 23 EPS gene clusters (strains CNRZ1066, CS8, EPS, S9, and SMQ-301). In the rest EPS clusters, *epsE* seems to encode a glycosyl-1-phosphate transferase. These enzymes initiate the assembly of the EPS repeating components through the transfer of phosphorylated sugars to the undecaprenyl-phosphate lipid carrier on the cytoplasmic side of the bacterial membrane (Broadbent et al., 2003; Wu et al., 2014). The sugar is transferred to the outer side of the membrane and this translocation process is probably facilitated by a flippase protein (Manat et al., 2014). All cluster A strains, including strain NCTC12958^T^, carried one flippase coding gene with the exception of strain ST3 which carried two. In contrast, all strains from cluster B seem to lack the respective gene with the exception of strain B59671.

The genes downstream *epsE* encode proteins with various functions related to EPS biosynthesis. Among them, glycosyltransferases are involved in the consecutive transport of nucleotide sugar moieties to the lipid carrier. Both the number and the type of the respective genes in the EPS clusters are variable and may influence the composition of the produced EPS (Cui et al., 2017). The current analysis revealed the presence of transferases commonly encountered in *S. thermophilus* EPS clusters, such as glucosyltransferases, galactosyltransferases, and rhamnosyltransferases. A UDP-galactopyranose mutase involved in the synthesis of UDP-galactofuranose was identified in half of the EPS clusters. Interestingly, only strains JIM 8232, GABA, and NCTC12958^T^ were found to carry a gene encoding a putative galactofuranosyl-transferase. Finally, genes implicated in the polymerization and translocation of the EPS repeating units have been also identified in all EPS clusters, as reported previously for *S. thermophilus* strains (Goh et al., 2011; Wu et al., 2014; Cui et al., 2017; Evivie et al., 2017).

Based on synteny, the EPS gene clusters can be categorized practically in distinct groups, supporting AI, AII, AIV, and BI subgroups. EPS clusters of strains KLDS 3.1003 and ST3 were highly similar to subgroup AI. Similarities in EPS clusters were also observed beyond lineages, as in the case of strains GABA and NCTC12958^T^. Certain EPS gene clusters, namely those of strains ACA-DC 2, LMD-9, LMG 18311, and B59671, presented higher structural variability due to the presence of many unique genes, which are coding mostly hypothetical proteins and glycosyltransferases. These observations are in accordance with previous findings for strains LMD-9 and LMG 18311 (Goh et al., 2011). Of note, three recent studies have been performed to highlight molecular mechanisms of EPS production in strains ASCC 1275 and KLDS SM (Li et al., 2018; Padmanabhan et al., 2018; Wu and Shah, 2018), while a fourth study suggests a protective role of purified EPS isolated from strain MN-BM-A01 against colitis in mice (Chen et al., 2019). The lineage like-patterns we observed among EPS gene clusters could potentially be useful for extrapolating findings from one strain to another. In all cases understanding of the EPS biosynthesis in *S. thermophilus* may allow a better selection of strains or even their engineering for improved dairy and probiotic products (Xiong et al., 2019a).

### Proteolytic System

The proteolytic system of LAB has been extensively investigated. A number of studies have revealed the diversity of its components, i.e., cell-wall bound proteinases, peptide and amino acid transporters and peptidases, among various LAB species (Savijoki et al., 2006; Liu et al., 2010). In the present study, the proteolytic system of *S. thermophilus* strains was examined on the basis of the scheme published by Liu et al. (2010) and the recent work of Tian et al. (2018). The results acquired from the TransportDB database were also employed.

Due to the limited availability of free amino acids and peptides in milk, the degradation of caseins is essential for growth. In *S. thermophilus*, the cell-wall associated proteinase PrtS is implicated in the initiation of the proteolytic cascade (Hols et al., 2005; Goh et al., 2011; Tian et al., 2018). *prtS* is present in almost half of the strains examined. The analysis showed that the respective gene is present (intact or truncated) solely in cluster A strains with the exception of strains APC151, KLDS 3.1003, and ND03 (Supplementary Table S5A). As it has been previously reported, PrtS presents 95% identity to the PrtS protein of *Streptococcus suis* and the distribution of *prtS* in *S. thermophilus* strains is infrequent in historical collections compared to industrial ones, indicating acquisition by lateral transfer in the species population (Delorme et al., 2010). PrtS has been related to the rapid growth of *S. thermophilus* in milk as a mono-culture and therefore in the rapid acidification of milk, which is a desirable technological trait. However, the sole presence of *prtS* is not sufficient for the rapid milk acidification by *S. thermophilus*. Milk acidification seems to be a complex phenotypic trait, which involves the overexpression of several genes (Galia et al., 2016). Furthermore, it was demonstrated that *S. thermophilus* strains, irrespective of the *prtS*^+/–^ status, may present cell-associated extracellular peptidase activities. These activities, albeit weaker than that of PrtS, could probably provide amino acids essential for *S. thermophilus* growth (Hafeez et al., 2015). The extracellular presence of PepX aminopeptidase in *S. thermophilus* was recently suggested (Hafeez et al., 2019). Nevertheless, it has been supported that only *prtS*^–^ *S. thermophilus* strains can perform protocooperation with *L. bulgaricus* (Settachaimongkon et al., 2014).

Several peptide and amino acid transporters of various families have been predicted in all *S. thermophilus* strains (Supplementary Table S5B). The majority of these transporters belong to the ATP-binding cassette (ABC) superfamily and include one oligopeptide Opp ABC transporter, one branched-chain amino acid ABC transporter, one glutamine ABC transporter, four amino acid ABC transporters, one spermidine/putrescine ABC transporter and one methionine ABC transporter. In a number of instances, the gene clusters of these transporters may contain putative pseudogenes and thus may be not functional. It has been previously reported that strain LMD-9 carries a second Opp ABC transporter, which is homologous to that of *Bifidobacterium* species (Goh et al., 2011). This transporter is also present in strains SMQ-301 and ST3. Strains B59671, GABA, and NCTC12958^T^ have one extra amino acid ABC transporter, which displays high identity (90%) with the respective one of *S. salivarius* (data not shown). Furthermore, all strains carry four amino acid permeases of the amino acid-polyamine-organocation (APC) family. Additionally, strains ACA-DC 2, APC151, B59671, GABA, KLDS 3.1003, and ND03 carry a glutamate/GABA antiporter (*gadC*) (Supplementary Table S5B). The latter gene along with glutamate decarboxylase gene (*gadB*) are responsible for gamma-aminobutyric acid (GABA) production. It was recently demonstrated that strain APC151 is a high-yield GABA producer (Linares et al., 2016). In strain KLDS 3.1003 a unique histidine/histamine antiporter has been also identified (*hdcP*) (Supplementary Table S5B). The respective gene is located adjacently to a unique histidine decarboxylase gene (*hdcA*) and along with *hdcB* form the *hdc* cluster, probably acquired by HGT (please see below) which has been previously described in two other strains of *S. thermophilus* (Calles-Enríquez et al., 2010). From a physiological point of view, this gene cluster is probably implicated in cell protection under acidic conditions (De Angelis and Gobetti, 2011). The use of histamine-producing *S. thermophilus* strains should be avoided in dairy manufacture, since it has been demonstrated that *hdcA*^+^ *S. thermophilus* used as starter in cheese production was associated with the accumulation of histamine in the final product (Gardini et al., 2012). One di-tripeptide transporter is present in all strains. A branched-chain amino acid permease and an amino efflux protein are also present in all strains, but for B59671 and ST3, respectively. The transport of the branched-chain amino acids leucine, isoleucine, and valine, as well as alanine, serine/threonine and glutamate/aspartate is probably facilitated by four symporters, three of them being present in all strains and only one in six strains (Supplementary Table S5B). In addition, a number of incomplete ABC transporters has been also predicted in all the strains analyzed (data not shown).

Besides PrtS, 12 highly conserved cytoplasmic peptidases have been identified in all strains, namely *pepA*, *pepC*, *pepF*, *pepM*, *pepN*, *pepO*, *pepP*, *pepQ*, *pepS*, *pepT*, *pepV*, and *pepX* (Supplementary Table S5A). Moreover, a number of peptidases, which have been identified in several LAB species, are missing from all *S. thermophilus* strains (Liu et al., 2010). More specifically, pyrrolidone-carboxylate peptidase (*pcp*) and proline peptidases *pepI*, *pepR*, and *pepL* are absent. Cysteine aminopeptidase (*pepE/pepG*) presents 40% identity with aminopeptidase C in all *S. thermophilus* strains, while a putative dipeptidase *pepD* is present but truncated in 14 *S. thermophilus* strains. It should be mentioned that the universal distribution of the majority of genes encoding proteins of the proteolytic system of *S. thermophilus* supports the essential role of the system.

### Amino Acids Biosynthesis

The *in silico* analysis of amino acid biosynthetic pathways has been addressed in *S. thermophilus* (Hols et al., 2005). Experimental data for the species have been acquired for the biosynthesis of proline, branched-chain amino acids, glutamine and aspartate (Limauro et al., 1996; Garault et al., 2000; Monnet et al., 2005; Arioli et al., 2007). Furthermore, Pastink et al. (2009) studied the amino acid metabolism and amino acid dependency of strain LMG 18311 through amino acid omission experiments, concluding that the minimal amino acid auxotrophy for the strain involves histidine and one of the sulfur-containing amino acids (methionine or cysteine). In some *S. thermophilus* strains amino acid requirements for growth involve at least four amino acids (Glu, Cys, His, and Met; Letort and Juillard, 2001). It seems that amino acid auxotrophy may be a strain dependent trait.

Most amino acid biosynthetic pathways are highly conserved in the 23 *S. thermophilus* strains (Supplementary Figure S4 and Supplementary Table S6). Analysis of *S. thermophilus* protein coding sequences, based on KEGG orthology assignments and Hols et al. (2005), revealed that the majority of the amino acid biosynthetic pathways are present in all strains examined. Complete biosynthetic pathways in all *S. thermophilus* strains were predicted for threonine, cysteine, glycine, proline, glutamine, asparagine, phenylalanine, alanine, aspartate, and glutamate. Current annotations of all *S. thermophilus* strains in Refseq with prokaryotic genome annotation pipeline (PGAP) do not seem to support biosynthesis of lysine due to the absence of *dapE*, *dapH*, and *dapF* (Supplementary Table S6). An incomplete Dap-pathway was also reported for strains LMG 18311 (Hols et al., 2005) and LMD-9 (Goh et al., 2011). However, experimental evidence suggests biosynthesis of lysine in strains LMG 18311 (Pastink et al., 2009) and MN-ZLW-002 (Qiao et al., 2018) presumably through a complete Dap-pathway. We found that this discrepancy may be an artifact of annotation with the PGAP tool. Older *S. thermophilus* GenBank files, annotated with tools other than PGAP included a locus with three genes, the second of which is identified as a (truncated) *dapE* (data not shown). In contrast, in the same locus, PGAP predicts a single gene corresponding to a putative M20 peptidase pseudogene (e.g., locus_tag Y1U_RS01580 in strain MN-ZLW-002). We also tested other annotation tools, like rapid annotation using subsystem technology (RAST; Aziz et al., 2008) and FGenesB (Solovyev and Salamov, 2011) that also supported a three-gene architecture in the same locus, suggesting that further investigation is required to resolve this matter.

The most striking difference in the biosynthesis of amino acids among *S. thermophilus* strains examined concerns histidine. Hols et al. (2005) reported absence of this gene cluster in strains CNR1066 and LMG 18311 but its presence in strain LMD-9. As mentioned above, the respective pathway is complete in strains of cluster A and strain NCTC12958^T^, while strains of cluster B carry only one related gene, namely *hisK* (Supplementary Table S6 and Figure 5B). Furthermore, several amino acid biosynthetic pathways seem to be incomplete in a number of strains. Analysis revealed that in strain B59671 several genes involved in amino acid biosynthesis are putative pseudogenes or absent compared to the other strains. In this strain glutamate, serine, methionine and tyrosine biosynthetic pathways may be non-functional. Concerning the rest of the strains analyzed, incomplete biosynthetic pathways have been identified for methionine in NCTC12958^T^ and ST3, arginine in MN-BM-A01, branched-chain amino acids in JIM 8232 and tryptophan in EPS (Supplementary Table S6).

In some cases, differences among genes involved in specific biosynthetic steps during amino acid biosynthesis have been also identified. In tryptophan biosynthesis, two adjacent genes, namely *aroG1* and *aroG2* (Hols et al., 2005), encoding 70% identical proteins, have been identified in all strains except for strains ST3, CNRZ1066, and CS8. The first strain carries only *aroG1*, while the last two only *aroG2*. These genes are involved in the first step of chorismate synthesis, an intermediate product during tryptophan biosynthesis. Concerning the biosynthesis of branched-chain amino acids, in all *S. thermophilus* genomes two *ilvD* genes have been identified; one belongs to the *ilvDBNC* operon, while the second is located remotely from the *ilvDBNC* locus and its functionality is yet to be studied (Hols et al., 2005). The *ilvD* within the operon is a putative pseudogene in most strains and it seems to be functional only in KLDS 3.1003, LMD-9, NCTC12958^T^, and SMQ-301. These observations need further experimental investigation.

### Urea Metabolism

*Streptococcus thermophilus* is perhaps the sole species among the dairy LAB with the ability to hydrolyze urea, a phenotypic trait, which affects adversely the milk acidification rate (Pernoud et al., 2004; Iyer et al., 2010). The urease gene cluster is highly conserved in all *S. thermophilus* strains analyzed and comprises 11 genes in the form of an operon of 8.2 kbp size (Supplementary Table S7). It includes the acid-activated *ureI* gene, the structural genes *ureABC*, the accessory genes *ureEFGD* and the genes encoding the cobalt/nickel uptake system *ureMQO* (or *cbiMQO*) (Mora et al., 2004; Iyer et al., 2010). The *ureI* gene is located upstream the structural genes and is coding a pH-dependent urea channel, which is probably activated for compensating the increase of the extracellular acidity. The *ureABC* genes are coding the three structural subunits of the enzyme, with *ureC* coding the large subunit and the remaining two genes coding the two smaller subunits (Ninova-Nikolova and Urshev, 2013). The auxiliary genes *ureEFGD* encode metallochaperones involved in nickel metallocenter biosynthesis and the delivery of nickel ions to the active site of the urease. More specifically, the urease apoenzyme forms a complex with the UreD, UreF, and UreG proteins, which is activated by the addition of nickel, bicarbonate and the metallochaperone UreE (Sujoy and Aparna, 2013). The *ureMQO* system is probably responsible for the translocation of nickel ions into the bacterial cell as indicated by functional analysis of the homologous genes in *S. salivarius* (Chen and Burne, 2003).

The physiological role of *S. thermophilus* urease has not been thoroughly evaluated. Although it is considered a response mechanism to acid stress, it has been demonstrated that urease is produced at low levels also at neutral pH (Mora et al., 2005). The ureolytic activity of *S. thermophilus* is probably related not only to the biosynthesis of essential amino acids, e.g., glutamine, but to the overall nitrogen metabolism of the species, with the expression of the *ure* operon depending on aspartate, glutamate, glutamine, and NH_3_ concentrations (Monnet et al., 2005; Arioli et al., 2007). However, the rather uncommon urease-negative phenotype has been also reported for *S. thermophilus* strains, indicating that urease activity may not hold a vital role in milk fermentation (Mora et al., 2002). Recently, spontaneous urease-deficient mutants of *S. thermophilus* were isolated from *S. thermophilus* populations deriving from industrial yogurt starters. The stability of the mutated phenotype was confirmed, providing promising results regarding the potential use of urease-deficient strains as starters in dairy fermentations (Ninova-Nikolova and Urshev, 2013). However, in a recent study employing urease deficient mutants it was suggested that urease activity is important for yogurt acidification and that its absence inhibits fermentation acceleration during protocooperation with *L. bulgaricus* (Yamauchi et al., 2019).

### CRISPR-Cas Systems

The CRISPR-Cas systems are defense mechanisms widely distributed in prokaryotes, providing acquired immunity against foreign genetic elements like viruses and plasmids (Horvath and Barrangou, 2010). This immunity mechanism has been extensively studied in *S. thermophilus*, providing information concerning the environmental adaptability and the anti-phage activity of this microorganism (Sapranauskas et al., 2011; Louis et al., 2017; Hao et al., 2018). In addition, in certain studies spacers within CRISPR arrays in *S. thermophilus* were employed for assessing diversity among strains of the species (Horvath et al., 2008; Delorme et al., 2017). As mentioned above, Delorme et al. (2017) reported that MLST and whole genome based phylogeny differed from those inferred by CRISPR analysis. Here we revisit clustering of *S. thermophilu*s strains based on CRISPR analysis in the context of complete genome sequences that allowed us further validation of the diversity scheme we propose in this study.

**FIGURE 7:**
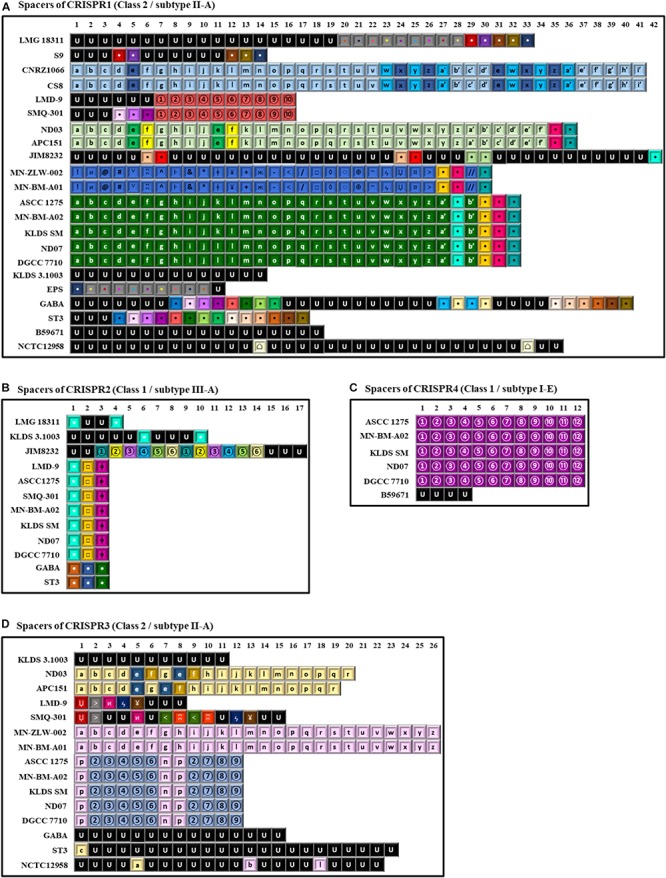
Spacer sequences alignment of the various clustered regularly interspaced short palindromic repeats-CRISPR associated (CRISPR-Cas) system types found in the 22 *S. thermophilus* strains. In the alignments only the spacer sequences have been used. In each type of CRISPR-Cas system each spacer is represented by the combination of a character and a font color. The spacers represented in black font with the letter U correspond to unique spacers. Spacers represented by the same combination of a character and a font color correspond to identical spacers. Spacers of CRISPR1 **(A)**, CRISPR2 **(B)**, CRISPR4 **(C)**, and CRISPR3 **(D)**.

As reported previously (Horvath and Barrangou, 2010), up to four distinct CRISPR-Cas loci, i.e., CRISPR1, CRISPR2, CRISPR3, and CRISPR4 were identified in our *S. thermophilus* strains (Supplementary Tables S8, S9A and Figure 7). CRISPR1 and CRISPR3 both belong to Class 2/subtype II-A CRISPR-Cas systems, while CRISPR2 and CRISPR4 belong to Class 1/subtype III-A and Class 1/subtype I-E CRISPR-Cas systems, respectively (Horvath et al., 2008; Makarova et al., 2015; Hao et al., 2018). Furthermore, one putative orphan CRISPR array structure was predicted by CRISPRFinder in strains JIM 8232 and LMG 18311, characterized by the absence of adjacent Cas proteins. The direct repeats (DRs) of this array in JIM 8232 were identical to the DRs of CRISPR3 in other strains, suggesting that it must have owned the relevant Cas proteins originally and subsequently lost them. In contrast, the DRs of LMG 18311 in the orphan array did not match any other DRs.

CRISPR1 was found in 22 out of the 23 *S. thermophilus* strains analyzed here, with ACA-DC 2 carrying no CRISPR array despite retaining CRISPR-related genes (Alexandraki et al., 2017). CRISPR1 array size ranged between 760 and 2,805 bp. This size variability is associated with the number of spacers (11–42) in the arrays of the different strains. This is the largest CRISPR array within the *S. thermophilus* strains analyzed with the exception of strain ST3 (Figure 7) and it has been reported to be ubiquitous in *S. thermophilus* strains (Horvath et al., 2008). In strains B59671 and KLDS 3.1003 the gene coding the Cas9 protein is a putative pseudogene, indicating that the respective CRISPR-Cas systems might have been inactivated. Strains ASCC 1275, APC151, DGCC 7710, GABA, KLDS 3.1003, KLDS SM, LMD-9, MN-BM-A02, MN-ZLW-002, ND03, ND07, NCTC12958^T^, SMQ-301, and ST3 also carry CRISPR3. This CRISPR contains 8 to 26 spacers and in most cases is shorter than CRISPR1 (Figure 7). A higher activity for CRISPR1 in comparison to CRISPR3 has been experimentally validated (Horvath et al., 2008). In the case of CRISPR3, *cas9* is a putative pseudogene in strain MN-BM-A01, indicating that the specific system may have been also inactivated. It should be emphasized that CRISPR1 is detected in both cluster A and B strains, while CRISPR3 is present only in cluster A (apart from strain JIM 8232) and it is totally absent from cluster B strains. Based on analysis of the LMD-9 genome sequence, Horvath et al. (2008) proposed that the entire CRISPR3-Cas system may have been deleted or inserted in *S. thermophilus* strains through a recombination event between a repeat present in the terminal repeat of CRISPR3 and a repeat close to *serB* which flanks the system from one side.

CRISPR2 was found in strains ASCC 1275, DGCC 7710, GABA, KLDS 3.1003, KLDS SM, JIM 8232, LMD-9, LMG 18311, MN-BM-A02, ND07, SMQ-301, and ST3. Thus, CRISPR2 was present only in cluster A strains, apart from strain LMG 18311 which belongs to cluster B. Among the Cas proteins of CRISPR2, *cas1* is a putative pseudogene in strains KLDS 3.1003 and LMG 18311, while *cas10* is a putative pseudogene in strains LMD-9 and SMQ-301. Furthermore, the respective CRISPR-Cas systems of strains GABA and ST3 carry only three CRISPR-associated genes (*cas1*, *cas2*, and *csm6*) which indicates that they are incomplete. All these CRISPR2 systems carried a CRISPR array. However, additional “possible” CRISPR2 systems were predicted by CRISPRFinder owning an incomplete set of Cas proteins followed by a single spacer within two DRs (Supplementary Table S9A). Our findings suggest inactivation and/or degeneration of CRISPR2 in several strains. Horvath et al. (2008) reported that CRISPR2 may indeed be inactivated in certain strains, however Tamulaitis et al. (2014) were able to demonstrate its activity in at least another strain. CRISPR4 was identified in strains ASCC 1275, B59671, DGCC 7710, KLDS SM, MN-BM-A02, and ND07. Genes *cse1* and *cas2* in strains ASCC 1275 and B59671, respectively, are putative pseudogenes. Interestingly, the CRISPR4 was basically found in subgroup AI strains. Further subgrouping could be supported not only through the presence/absence of CRISPR-Cas systems, but also through the distribution of different spacers, as discussed below.

A total of 997 spacers were found in the confirmed CRISPR-Cas systems of the 22 *S. thermophilus* strains with 93% being assigned in CRISPR1 and CRISPR3. Analysis of the respective sequences revealed that 258 are unique among 11 strains, namely NCTC12958^T^, JIM 8232, GABA, KLDS 3.1003, ST3, B59671, LMG 18311, LMD-9, SMQ-301, S9, and EPS, while 253 appeared more than once in the CRISPR arrays. As shown previously, CRISPR arrays may be employed for accessing strain diversity within *S. thermophilus* (Horvath et al., 2008; Delorme et al., 2017). Indeed, looking into the architecture of the CRISPR arrays we could identify once more patterns that are not shared by all *S. thermophilus* strains, but they are specific to the grouping of strains we have already described. For example, CRISPR1 supports subgroups AI, AII, AIII, and AIV. Subgroup BI is partially supported, since only strains CNRZ1066 and CS8 share the same CRISPR array. CRISPR3 supports subgroups AI, AII, AIII, and AIV. CRISPR4 has a unique pattern of spacers for subgroup AI. As mentioned above CRISPR2 is present only in cluster A strains, apart from strain LMG 18311 which belongs to cluster B, but the spacer pattern in the arrays could not distinguish any subgroup (Figure 7). Most spacers were unique for each subgroup and were present in a specific order in the array. This observation suggests that this part of the array was present in the common ancestor of these subgroups of strains. However, in certain instances, a specific spacer could be found common between two seemingly unrelated arrays belonging to different subgroups of strains. Most probably such spacers were acquired by the common ancestor of each subgroup due to exposure to the same exogenous DNA that resulted in the acquisition of the same part of sequence into the specific CRISPR array. Evidently, these spacers were identified only in arrays of the same class and subtype CRISPR-Cas systems. Similar analysis of spacers to infer evolutionary relationships among *S. thermophilus* strains have been reported previously (Horvath et al., 2008). However, when looking solely to the architecture of the CRISPR array it is very difficult to distinguish between clones or complexes of very similar strains that are not actual clones.

BLASTN analysis of the spacers showed that 317 sequences matched several different *S. thermophilus* bacteriophages (Supplementary Table S9B). Almost half of the spacers analyzed could be related to phages 7201, Sfi19, Sfi21, DT1, and Sfi11. This finding may indicate a high frequency of exposure of *S. thermophilus* to the specific phages. Finally, six spacers were highly identical to *Lactococcus* phages, while 12 spacers were highly identical to plasmids of *Enterococcus faecium*, *S. suis*, *Streptococcus pyogenes*, *S. pneumoniae*, *Lactobacillus salivarius* and *Lactococcus lactis*. These findings indicate that *S. thermophilus* has been found in the same environment with these bacteria. Furthermore, it could be hypothesized that at least some potential HGT events of plasmid donation toward *S. thermophilus* were aborted through the activity of CRISPR-Cas systems. Overall our findings are in agreement with previous results (Bolotin et al., 2005; Horvath et al., 2008).

### R-M Systems and Prophages

Another immunity mechanism employed by the prokaryotes against foreign DNA are the R-M systems. All *S. thermophilus* strains analyzed carry several R-M systems, classified into four types (Roberts et al., 2005, 2015; Supplementary Table S10 and Supplementary Figure S5). The majority of strains carry one complete type I R-M system with strains EPS, NCTC12958^T^, GABA, and KLDS 3.1003 carrying two. No type I R-M system was predicted for strain B59671, while in strains MN-ZLW-002, MN-BM-A01, and ST3 the predicted type I R-M system was incomplete due to the absence or inactivation of one or more of the necessary genes. This was the case for additional predicted type I systems in several strains. Certain *S. thermophilus* strains carry at least one type II system with strains LMD-9, MN-BM-A01, ND03, and APC151 owning three such systems. Unlike type I R-M systems, most type II systems seem to be complete and potentially active. One type III system is present in strains ACA-DC 2, CNRZ1066, CS8, EPS, S9, LMG 18311, NCTC12958^T^, and GABA. Finally, a type IV system has been predicted in almost half of the strains analyzed, which contains only a restriction enzyme that recognizes and cuts modified DNA.

A more detailed investigation revealed that type II and type III R-M systems are absent or inactivated from *S. thermophilus* strains of subgroup AI. For this reason, we wanted to examine whether the presence/absence pattern of R-M systems in *S. thermophilus* is lineage specific. As demonstrated in Supplementary Figure S5 in several instances R-M systems are distributed on the chromosome in a manner that is characteristic for a potential lineage. This is particularly obvious for cluster A and more specifically for subgroups I, II, III, and IV. The R-M systems of strains in cluster B presented some similarities within the same subgroup, but they were more variable.

Despite the presence of the aforementioned defense mechanisms, complete prophages were also predicted for strains APC151, ND03, and NCTC12958^T^, while in the rest of the examined genomes only remnants of prophages have been identified (data not shown). In strains APC151 and ND03 the same prophage was predicted located within the EPS cluster of each strain. In strain NCTC12958^T^ the intact prophage was previously described as phage 20617 by Arioli et al. (2018). Interestingly, the authors of this study demonstrated that the lysogenic strain NCTC12958^T^ (DSM 20617^T^) exhibited higher adhesion to solid surfaces and heat resistance compared to the phage-cured derivative strain, suggesting some competitive advantage due to the stable association of the phage and the host.

### Genomic Islands

Genomic islands acquired through HGT can provide adaptive and technological traits to the host microorganism (Juhas et al., 2009). *In silico* prediction of HGT in *S. thermophilus* has been previously reported (Hols et al., 2005; Liu et al., 2009; Eng et al., 2011). In this study, the GIs predicted by IslandViewer 4 in *S. thermophilus* ranged from 5 to 23 per strain, with sizes between 3.5 and 58 kbp and variable GC content from 26.1 to 45.2% (Supplementary Table S11). A total of 253 GIs were predicted, 31 of which were unique in 11 strains. The rest of the GIs have been identified in at least two *S. thermophilus* strains, either complete or partial. Of note, the genome array of ribosomal proteins was predicted as part of a GI in a number of strains. This is a false positive result, since it has been reported that the nucleotide composition of these arrays differentiates significantly from the rest protein coding genes (Hols et al., 2005; Fernandez-Gomez et al., 2012). Thus, these GIs (14 in total) were excluded from further analysis (Supplementary Tables S11, S12A). Several GIs were found to be present in both clusters A and B strains, while others were present either in cluster A or B strains. The first type of GIs was most probably acquired earlier than the second, i.e., before clusters A and B were separated. In accordance with what has been reported above for other genomic features, certain subgroups of strains display a unique distribution pattern of specific GIs that can support subgroups AI–AIV and BI (Supplementary Figure S6).

BLASTN analysis of the predicted GIs could not always reveal a potential donor. Nevertheless, a number of GIs could be traced back to specific microorganisms (coverage >70%, identity >90%; Supplementary Table S12B). The majority of species acting as potential donors belongs to the *Streptococcus* genus but also to other LAB like *L. lactis*, *Lactobacillus casei*, and *Leuconostoc gelidum*. In these last three cases GIs present high identity to plasmids carried by these organisms. In detail, specific GIs in subgroups AI, AII, and one GI in strain NCTC12958^T^ present high identity to plasmids pLd7/p229C of *L. lactis* subsp. *lactis* (Kelleher et al., 2017; Van Mastrigt et al., 2018), pBD-II/pLC2W of *L. casei* (Ai et al., 2011; Chen et al., 2011; Song et al., 2018) and plasmid 1 of *L. gelidum* subsp. *gasicomitatum* (Andreevskaya et al., 2016), respectively. It is interesting to highlight that strains of *S. thermophilus* seem to have also interacted with members of the *Streptococcus bovis*/*Streptococcus equinus* complex (SBSEC), namely *S. macedonicus*, *S. infantarius* subsp. *infantarius*, *Streptococcus gallolyticus*, and *S. equinus*. Members of the complex are established members of the gastrointestinal tract (GIT) of ruminants, while certain species like *S. macedonicus* and *S. infantarius* are increasingly associated with fermented foods, especially of dairy origin (Jans et al., 2013a, b; Papadimitriou et al., 2014, 2015a).

A detailed investigation of the annotated features of *S. thermophilus* GIs revealed that they could be involved in EPS biosynthesis in accordance with previous findings reported for strains CNRZ1066, LMD-9, and LMG 18311 (Liu et al., 2009). CRISPR-Cas and complete R-M systems have been also identified in GIs. This would include CRIPR3 and CRISPR4 and type I and III R-M systems. In addition, the 38.5 kbp GI 9 contains most part of the intact prophage in strain NCTC12958^T^ (Supplementary Table S12A). Our analysis supports the presence of bacteriocin coding genes in the GIs of a number of strains. However, Hols et al. (2005) suggested that the activity of these antimicrobial peptides may not be always guaranteed due to the absence of genes coding for transport or immunity proteins or other differences. For example, the locus of a class II bacteriocin-like peptide (*blp*) was experimentally studied in strains CNRZ1066, LMG 18311, and LMD-9 and it was concluded that it is only functional in the last strain (Hols et al., 2005). In strain B59671, GI 5 carries genes of the *blp* gene cluster involved in the production of the bacteriocin thermophilin 110 (Renye et al., 2017). Finally, in GI 6 of strain GABA we found a locus containing several genes coding for leader peptides (including mutacin IV, BlpU, and bovicin 255), but transport or immunity proteins seem to be inactive or absent (Supplementary Table S12A). Moreover, several genes involved in amino acid transport have been found in the predicted GIs of *S. thermophilus* strains. Some of these include a glutamate:GABA antiporter in strains APC151, GABA, and ND03, a dicarboxylate/amino acid:cation symporter in strains APC151, KLDS 3.1003, MN-BM-A01, MN-ZLW-002, ND03, and ST3 and a complete amino acid ABC transporter in strains CS8, EPS, KLDS 3.1003, and S9. The *hdc* cluster of strain KLDS 3.1003 was also identified in a GI and BLASTN analysis revealed possible HGT from a satellite phage. Furthermore, GI 7 of strain JIM 8232 corresponds to the biosynthetic gene cluster of histidine. As already mentioned, this region is also present in all cluster A *S. thermophilus* strains (plus strain NCTC12958^T^) but for unknown reasons it was assigned as a GI only in JIM 8232. BLASTN analysis revealed that this region presents high identity to the SBSEC member *S. equinus* (92%) supporting its potential acquisition by HGT in *S. thermophilus* chromosome. In addition, genes involved in fatty acid biosynthesis were identified in GIs of strains APC151, GABA, MN-BM-A01, MN-ZLW-002, and ND03, while stress response genes, e.g., coding for cold-shock proteins were also identified in a number of strains, including ASCC 1275, CNRZ1066, KLDS 3.1003, LMG 18311, ND03, and ST3. Finally, the gene cluster *cbs*-*cblB*-*cysE* involved in the metabolism of sulfur-containing amino acids has been previously suggested to have been transmitted by HGT from *L. bulgaricus* or *Lactobacillus helveticus* to *S. thermophilus* (Liu et al., 2009). Current analysis revealed that the respective cluster was predicted as part of a bigger GI in 17 *S. thermophilus* strains. More specifically, this GI along with the three genes were identified in strains APC151, GABA, KLDS 3.1003, LMD-9, LMG 18311, MN-BM-A01, MN-ZLW-002, ND03, and SMQ-301, while in strains ACA-DC 2, ASCC 1275, CNRZ1066, CS8, MN-BM-A02, ND07, S9, and ST3 the *cysE* is a putative pseudogene (Supplementary Table S12A).

It should be mentioned that Selle et al. (2015) identified four expendable GIs in the genome of strain LMD-9 with variable distribution in other sequenced strains. IslandViewer 4 did not predict GIs 1 and 2 reported in that study, while it detected GIs overlapping or included in GIs 3 and 4. These differences can be explained by the *in silico* methods employed to detect GIs. Selle et al. (2015) employed a strategy combining the location of potentially essential open reading frames (ORFs) and highly similar insertion sequences (ISs) which is distinct from the strategies employed by the tools included in IslandViewer 4.

### *S. thermophilus* Genes Implicated in Protocooperation With *L. bulgaricus*

The bacterial pair of *S. thermophilus* and *L. bulgaricus* is routinely employed in yogurt production. The mutually beneficial interaction between these bacteria in the yogurt ecosystem, known as protocooperation, is based on the exchange of metabolites and results in improved metabolic performance related to accelerated acidification, enhanced EPS production and abundance of aroma volatiles. Initially, *S. thermophilus* boosts the growth of *L. bulgaricus* by lowering the pH and providing formic, pyruvic and folic acid as well as carbon dioxide. Subsequently, *L. bulgaricus* stimulates *S. thermophilus* growth by producing peptides and free amino acids (Settachaimongkon et al., 2014). Transcriptome analysis of a mixed *S. thermophilus* and *L. bulgaricus* culture also supports that metabolites like formic and folic acid produced by *S. thermophilus* are utilized by *L. bulgaricus* as precursors in purine biosynthesis (Sieuwerts et al., 2010). *S. thermophilus* carries genes encoding pyruvate formate lyase (PFL) and pyruvate formate-lyase activating (PFLA) enzyme, while *L. bulgaricus* lacks these genes (Nishimura et al., 2013). Our analysis revealed the presence of both *pfl* and *pflA* in all *S. thermophilus* strains examined (Supplementary Table S13).

In addition, a number of studies have been performed concerning the role of PrtS produced by *S. thermophilus* during manufacture of dairy products, especially yogurt. For example, PrtS production may positively affect *S. thermophilus* growth in a pure culture, but it may be neutral in a mixture with *L. bulgaricus* strains producing the protease PrtB (Courtin et al., 2002). In a more recent study, it was demonstrated that only non-proteolytic *S. thermophilus* strains performed protocooperation with *L. bulgaricus* (Settachaimongkon et al., 2014). As already mentioned, the majority of cluster A strains carries *prtS*, while it is absent from all cluster B strains, indicating that the latter may be more appropriate for protocooperation. However, specific *S. thermophilus* strains carrying the *prtS* have been shown to exhibit weak or no PrtS activity (Galia et al., 2009; Cui et al., 2016). In our dataset in strains MN-BM-A01 and SMQ-301 *prtS* was found to be truncated, an observation that may support to a degree the findings by Galia et al. (2009). Furthermore, it was recently reported that *prtS*^+^ strains may also present some technological advantages (Tian et al., 2018). We thus believe that more research is needed to establish the actual role of *prtS* regarding protocooperation.

The response of *S. thermophilus* to H_2_O_2_ produced by *L. bulgaricus* has also been studied. It appears that there is an inverse correlation between iron intake by *S. thermophilus* and H_2_O_2_ production by *L. bulgaricus*, and that *S. thermophilus* in the presence of H_2_O_2_ is regulating iron metabolism in order to diminish the production of harmful reactive oxygen species (ROS) (Herve-Jimenez et al., 2009; Sieuwerts et al., 2010). However, the results of two different studies are rather diverge. In one study, the expression patterns of *S. thermophilus* genes related to iron transport in the presence of *L. bulgaricus* were found to be upregulated (Sieuwerts et al., 2010), while in another study downregulated (Herve-Jimenez et al., 2009). Only *dpr* (peroxide resistance protein) and *fur* (ferric transport regulator protein) were found upregulated in both studies. *In silico* analysis of the 23 *S. thermophilus* strains revealed that *dpr* and *fur* belong to the core genome, while the iron ABC transporter is absent from strains JIM 8232, MN-ZLW-002, ND03, APC151, MN-BM-A01, and ST3 (Supplementary Table S13).

A novel protocooperation relationship between *S. thermophilus* and *L. bulgaricus* in yogurt fermentation concerns the bi-functional glutathione (GSH) synthetase gene of *S. thermophilus*, which produces GSH (Wang et al., 2016). The respective gene was found to be conserved in all 23 *S. thermophilus* strains analyzed (Supplementary Table S13). In a recent study, it was demonstrated that GSH produced by *S. thermophilus* provided protection to both *S. thermophilus* and *L. bulgaricus* cells toward acid stress. Additionally, the secreted GSH could enhance the growth of *L. bulgaricus* (Wang et al., 2016). Finally, genes related to EPS production were found to be upregulated in both microorganisms in a mixed culture when compared to monocultures, and thus they may play an important role in the texture of the final product (Sieuwerts et al., 2010). Given the heterogeneity observed in the EPS gene cluster of *S. thermophilus* strains, no mechanistic insight could be inferred.

## Conclusion

**FIGURE 8:**
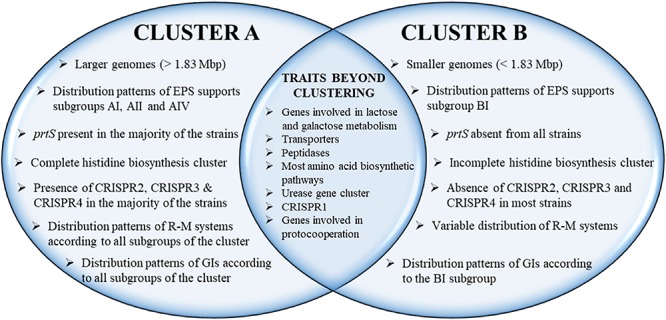
Schematic representation of structural and functional genomic traits that support the distinction of *S. thermophilus* strains in clusters A and B. The genomic features included in the common area of the Venn diagram are either present to all strains or they exhibit a presence/absence pattern beyond clusters A and B.

*Streptococcus thermophilus* is a starter of great economic significance for the dairy industry contributing to the production of world-wide consumed dairy products like yogurt and cheeses. A number of studies have been published in an attempt to explore and interpret various features of the species biology related to its technological potential. This became more feasible during the last two decades with the sequencing of genomes of *S. thermophilus* strains. In this study we analyzed 23 fully sequenced genomes of *S. thermophilus* in order to examine features of the species related to technological and evolutionary traits. Even from the beginning of our study, it became evident that strains of *S. thermophilus* present some variability considering the properties of the genomes (e.g., size, gene content, % of pseudogenes, rRNA and tRNA content). Core genome and ANI phylogenetic analysis revealed a specific pattern of clustering of strains (Figure 8). A main observation was that most strains could be separated in two major clusters. Cluster A was characterized by larger genomes, the presence of *prtS* in the majority of strains, the inclusion of a histidine biosynthesis gene cluster, as well as the presence of certain CRISPR-Cas system types and specific GIs. Strains in cluster B diversified from those in cluster A in all these aspects. These observations indicated the existence of at least two major lineages in *S. thermophilus* that appear at ANI values >98%. Further investigation suggested the presence of subgroups within the two clusters, i.e., subgroups AI–AIV and BI. The existence of these subgroups was also supported to a variable degree during COG analysis as well as the presence/absence pattern of specific loci and/or their organization, i.e., EPS clusters, CRISPR arrays, R-M systems and GIs. Clustering of *S. thermophilus* strains based on the spacers of CRISPR arrays has been performed before (Horvath et al., 2008; Delorme et al., 2017). Given the fact that CRISPR arrays can provide a retrospective view of the history of each strain based on the parasitic DNA it was exposed to, spacer sequences of the CRISPR1 which is present practically in all strains support the existence of evolutionary distinct lineages in *S. thermophilus*. Biodiversity within strains of *S. thermophilus* has been previously suggested using CRISPR array and/or MLST clustering (Horvath et al., 2008; Delorme et al., 2010, 2017; Yu et al., 2015). In our opinion, clustering of strains according to CRISPR array architecture or even MLST has important advantages (e.g., the ability to screen many strains), but these approaches may derive more easily to the characterization of potential clonal strains due to the use of limited genomic information. In contrast, whole genome phylogeny based on core genes should be more robust, while analysis of complete genome sequences may provide even more information concerning the discrimination of strains based on loci beyond core genome, like accessory genes or even unique genes. The subgroups we describe appeared at ANI values well above 99%, an observation that could indicate that they derive from clonal strains. A closer investigation of the data presented in this study suggests in some cases differences among strains of the same subgroup. For example, this becomes obvious when considering the exact sizes of the chromosome of the strains, the exact gene content (including accessory genes but also genes that are exclusively absent from a specific strain). In some instances, differences were observed in the EPS clusters, the distribution of R-M systems and GIs of strains within the same subgroup. Even though the differences among strains of the same subgroup may be rather subtle thus justifying the high ANI values at which their relatedness appears, they diversify strains beyond the strict definition of clones. Our analysis concerning the genome assemblies of the strains suggested a quality level that may not interfere with the grouping scheme we describe. Nonetheless, apart from the differences identified among the strains, our analysis also validated common features or features beyond the clustering pattern mentioned above (Figure 8). These would include characteristic traits for the adaptation of *S. thermophilus* to milk, like the conserved *gal-lac* and urease operons, the extended arsenal of peptidases and amino acid/peptide transporters in parallel to genes related to protocooperation. The high percentage of pseudogenes has been related to the reductive evolution of *S. thermophilus* during adaptation to rich in nutrients dairy niches (Bolotin et al., 2004; Hols et al., 2005; Goh et al., 2011). This trait was also apparent in all strains analyzed here. Interestingly, features related to milk adaptation seem to be also present in APC151. The strain does not diversify from the dairy strains, even though it was the only strain in our dataset that was isolated from a non-dairy environment, i.e., the fish intestine. This was also suggested previously (Linares et al., 2017). This relatively odd observation highlights the need to study strains found in environments different than milk and dairy products to fully apprehend the evolution of the species. Finally, the pan genome of the species is not closed yet, suggesting that sequencing of additional strains will be important. Certain new complete genomes have appeared in the databases since the initiation of our analysis (Proust et al., 2018; Renye et al., 2019), but more are required to further expand and validate any lineage-like patterns that may exist and could be related to the technological/probiotic repertoire of *S. thermophilus*.

## Data Availability Statement

All datasets generated for this study are included in the article/Supplementary Material.

## Author Contributions

VA and MK performed genome analysis and participated in the writing of the manuscript. JB and BP performed genome analysis. KP conceived the project, performed genome analysis, and participated in the writing of the manuscript. ET conceived the project and participated in the writing of the manuscript. All authors read and approved the final manuscript.

## Conflict of Interest

The authors declare that the research was conducted in the absence of any commercial or financial relationships that could be construed as a potential conflict of interest.

## Footnotes

1 http://genskew.csb.univie.ac.at/

2 https://plot.ly

3 http://www.rstudio.org

## Abbreviations

ABC: ATP-binding cassette
ANI: average nucleotide identity
APC: amino acid-polyamine-organocation
blp: bacteriocin-like peptide
BPGA: bacterial pan genome analysis
COG: clusters of orthologous groups
CRISPR-Cas: clustered regularly interspaced short palindromic repeats-CRISPR associated
DRs: direct repeats
EFSA: European Food Safety Authority
EPS: exopolysaccharide
FDA: Food and Drug Administration
GABA: gamma-aminobutyric acid
GIs: genomic islands
GIT: gastrointestinal tract
GRAS: generally recognized as safe
GSH: glutathione
HGT: horizontal gene transfer
ISs: insertion sequences
KEGG: Kyoto Encyclopedia of Genes and Genomes
KOALA: KEGG orthology and links annotation
LAB: lactic acid bacteria
LCBs: local collinear blocks
MiGA: microbial genomes atlas
MLST: multilocus sequence typing
ORFs: open reading frames
PFGE: pulsed-field gel electrophoresis
PFL: pyruvate formate lyase
PFLA: pyruvate formate-lyase activating
PGAP: prokaryotic genome annotation pipeline
QPS: qualified presumption of safety
RAPD: random amplification of polymorphic DNA
RAST: rapid annotation using subsystem technology
R-M: restriction-modification
ROS: reactive oxygen species
SBSEC: *Streptococcus bovis*/*Streptococcus equinus* complex.

## References

[B1] Ai L. Chen C. Zhou F. Wang L. Zhang H. Chen W. (2011). Complete genome sequence of the probiotic strain *Lactobacillus casei* BD-II. *J. Bacteriol.* 193 3160–3161. 10.1128/JB.00421-11 21478345 PMC3133187

[B2] Albertsen M. Hugenholtz P. Skarshewski A. Nielsen K. L. Tyson G. W. Nielsen P. H. (2013). Genome sequences of rare, uncultured bacteria obtained by differential coverage binning of multiple metagenomes. *Nat. Biotechnol.* 31 533–538. 10.1038/nbt.2579 23707974

[B3] Alexandraki V. Kazou M. Blom J. Pot B. Tsakalidou E. Papadimitriou K. (2017). The complete genome sequence of the yogurt isolate *Streptococcus thermophilus* ACA-DC 2. *Stand. Genomic Sci.* 12:18. 10.1186/s40793-017-0227-5 28163827 PMC5282782

[B4] Anbukkarasi K. Nanda D. K. Umamaheswari T. Hemalatha T. Singh P. Singh R. (2014). Assessment of expression of Leloir pathway genes in wild-type galactose-fermenting *Streptococcus thermophilus* by real-time PCR. *Eur. Food Res. Technol.* 239 895–903. 10.1007/s00217-014-2286-9

[B5] Anbukkarasi K. Umamaheswari T. Hemalatha T. Nanda D. K. Singh P. Rashmi H. M. (2013). Production of low browning Mozzarella cheese: screening and characterization of wild galactose fermenting *Streptococcus thermophilus* strains. *Int. J. Adv. Res.* 1 83–96.

[B6] Andreevskaya M. Hultman J. Johansson P. Laine P. Paulin L. Auvinen P. (2016). Complete genome sequence of *Leuconostoc gelidum* subsp. *gasicomitatum* KG16-1, isolated from vacuum-packaged vegetable sausages. *Stand. Genomic Sci.* 11:40. 10.1186/s40793-016-0164-8 27274361 PMC4895993

[B7] Arioli S. Eraclio G. Della Scala G. Neri E. Colombo S. Scaloni A. (2018). Role of temperate bacteriophage ϕ20617 on *Streptococcus thermophilus* DSM 20617T autolysis and biology. *Front. Microbiol.* 9:2719. 10.3389/fmicb.2018.02719 30473689 PMC6237837

[B8] Arioli S. Monnet C. Guglielmetti S. Parini C. De Noni I. Hogenboom J. (2007). Aspartate biosynthesis is essential for the growth of *Streptococcus thermophilus* in milk, and aspartate availability modulates the level of urease activity. *Appl. Environ. Microbiol.* 73 5789–5796. 10.1128/AEM.00533-07 17660309 PMC2074928

[B9] Arndt D. Grant J. R. Marcu A. Sajed T. Pon A. Liang Y. (2016). PHASTER: a better, faster version of the PHAST phage search tool. *Nucleic Acids Res.* 44 W16–W21. 10.1093/nar/gkw387 27141966 PMC4987931

[B10] Aziz R. K. Bartels D. Best A. A. Dejongh M. Disz T. Edwards R. A. (2008). The RAST Server: rapid annotations using subsystems technology. *BMC Genomics* 9:75. 10.1186/1471-2164-9-75 18261238 PMC2265698

[B11] Bai Y. Sun E. Shi Y. Jiang Y. Chen Y. Liu S. (2016). Complete genome sequence of *Streptococcus thermophilus* MN-BM-A01, a strain with high exopolysaccharides production. *J. Biotechnol.* 224 45–46. 10.1016/j.jbiotec.2016.03.003 26956372

[B12] Bertelli C. Laird M. R. Williams K. P. Simon Fraser University Research Computing Group, Lau B. Y. (2017). IslandViewer 4: expanded prediction of genomic islands for larger-scale datasets. *Nucleic Acids Res.* 45 W30–W35. 10.1093/nar/gkx343 28472413 PMC5570257

[B13] Blom J. Kreis J. Spanig S. Juhre T. Bertelli C. Ernst C. (2016). EDGAR 2.0: an enhanced software platform for comparative gene content analyses. *Nucleic Acids Res.* 44 W22–W28. 10.1093/nar/gkw255 27098043 PMC4987874

[B14] Bolotin A. Quinquis B. Renault P. Sorokin A. Ehrlich S. D. Kulakauskas S. (2004). Complete sequence and comparative genome analysis of the dairy bacterium *Streptococcus thermophilus*. *Nat. Biotechnol.* 22 1554–1558. 10.1038/nbt1034 15543133 PMC7416660

[B15] Bolotin A. Quinquis B. Sorokine A. Dusko Ehrlich S. (2005). Clustered regularly interspaced short palindrome repeats (CRISPRs) have spacers of extrachromosomal origin. *Microbiology* 151 2551–2561. 10.1099/mic.0.28048-0 16079334

[B16] Broadbent J. R. Mcmahon D. J. Welker D. L. Oberg C. J. Moineau S. (2003). Biochemistry, genetics, and applications of exopolysaccharide production in *Streptococcus thermophilus*: a review. *J. Dairy Sci.* 86 407–423. 10.3168/jds.S0022-0302(03)73619-4 12647947

[B17] Burall L. S. Grim C. J. Mammel M. K. Datta A. R. (2016). Whole genome sequence analysis using JSpecies tool establishes clonal relationships between *Listeria monocytogenes* strains from epidemiologically unrelated listeriosis outbreaks. *PLoS One* 11:e0150797. 10.1371/journal.pone.0150797 26950338 PMC4780826

[B18] Calles-Enríquez M. Eriksen B. H. Andersen P. S. Rattray F. P. Johansen A. H. Fernández M. (2010). Sequencing and transcriptional analysis of the *Streptococcus thermophilus* histamine biosynthesis gene cluster: factors that affect differential *hdcA* expression. *Appl. Environ. Microbiol.* 76 6231–6238. 10.1128/AEM.00827-10 20656875 PMC2937487

[B19] Chaudhari N. M. Gupta V. K. Dutta C. (2016). BPGA- an ultra-fast pan-genome analysis pipeline. *Sci. Rep.* 6:24373. 10.1038/srep24373 27071527 PMC4829868

[B20] Chen C. Ai L. Zhou F. Wang L. Zhang H. Chen W. (2011). Complete genome sequence of the probiotic bacterium *Lactobacillus casei* LC2W. *J. Bacteriol.* 193 3419–3420. 10.1128/JB.05017-11 21515769 PMC3133282

[B21] Chen Y. Zhang M. Ren F. (2019). A role of exopolysaccharide produced by *Streptococcus thermophilus* in the intestinal inflammation and mucosal barrier in Caco-2 monolayer and dextran sulphate sodium-induced experimental murine colitis. *Molecules* 24:513. 10.3390/molecules24030513 30708992 PMC6384629

[B22] Chen Y.-Y. M. Burne R. A. (2003). Identification and characterization of the nickel uptake system for urease biogenesis in *Streptococcus salivarius* 57.I. *J. Bacteriol.* 185 6773–6779. 10.1128/jb.185.23.6773-6779.2003 14617641 PMC262724

[B23] Courtin P. Monnet V. Rul F. (2002). Cell-wall proteinases PrtS and PrtB have a different role in *Streptococcus thermophilus*/*Lactobacillus bulgaricus* mixed cultures in milk. *Microbiology* 148 3413–3421. 10.1099/00221287-148-11-3413 12427933

[B24] Cui Y. Jiang X. Hao M. Qu X. Hu T. (2017). New advances in exopolysaccharides production of *Streptococcus thermophilus*. *Arch. Microbiol.* 199 799–809. 10.1007/s00203-017-1366-1 28357474

[B25] Cui Y. Xu T. Qu X. Hu T. Jiang X. Zhao C. (2016). New insights into various production characteristics of *Streptococcus thermophilus* strains. *Int. J. Mol. Sci.* 17:E1701. 10.3390/ijms17101701 27754312 PMC5085733

[B26] Darling A. E. Mau B. Perna N. T. (2010). progressiveMauve: multiple genome alignment with gene gain, loss and rearrangement. *PLoS One* 5:e11147. 10.1371/journal.pone.0011147 20593022 PMC2892488

[B27] Darling A. E. Miklós I. Ragan M. A. (2008). Dynamics of genome rearrangement in bacterial populations. *PLoS Genet.* 4:e1000128. 10.1371/journal.pgen.1000128 18650965 PMC2483231

[B28] De Angelis M. Gobetti M. (2011). “Stress responses of lactobacilli,” in *Stress Responses of Lactic Acid Bacteria*, eds Tsakalidou E. Papadimitriou K., (New York, NY: Springer Science+Business Media), 219–249. 10.1007/978-0-387-92771-8_11

[B29] De Vin F. Radstrom P. Herman L. De Vuyst L. (2005). Molecular and biochemical analysis of the galactose phenotype of dairy *Streptococcus thermophilus* strains reveals four different fermentation profiles. *Appl. Environ. Microbiol.* 71 3659–3667. 10.1128/AEM.71.7.3659-3667.2005 16000774 PMC1168995

[B30] Degeest B. De Vuyst L. (2000). Correlation of activities of the enzymes alpha-phosphoglucomutase, UDP-galactose 4-epimerase, and UDP-glucose pyrophosphorylase with exopolysaccharide biosynthesis by *Streptococcus thermophilus* LY03. *Appl. Environ. Microbiol.* 66 3519–3527. 10.1128/aem.66.8.3519-3527.2000 10919816 PMC92180

[B31] Delorme C. Abraham A. L. Renault P. Guédon E. (2015). Genomics of *Streptococcus salivarius*, a major human commensal. *Infect. Genet. Evol.* 33 381–392. 10.1016/j.meegid.2014.10.001 25311532

[B32] Delorme C. Bartholini C. Bolotine A. Ehrlich S. D. Renault P. (2010). Emergence of a cell wall protease in the *Streptococcus thermophilus* population. *Appl. Environ. Microbiol.* 76 451–460. 10.1128/AEM.01018-09 19915034 PMC2805209

[B33] Delorme C. Bartholini C. Luraschi M. Pons N. Loux V. Almeida M. (2011). Complete genome sequence of the pigmented *Streptococcus thermophilus* strain JIM8232. *J. Bacteriol.* 193 5581–5582. 10.1128/JB.05404-11 21914889 PMC3187418

[B34] Delorme C. Legravet N. Jamet E. Hoarau C. Alexandre B. El-Sharoud W. M. (2017). Study of *Streptococcus thermophilus* population on a world-wide and historical collection by a new MLST scheme. *Int. J. Food Microbiol.* 242 70–81. 10.1016/j.ijfoodmicro.2016.11.016 27894009

[B35] Dupuis M.-È. Villion M. Magadán A. H. Moineau S. (2013). CRISPR-Cas and restriction–modification systems are compatible and increase phage resistance. *Nat. Commun.* 4:2087. 10.1038/ncomms3087 23820428

[B36] Edgar R. C. (2010). Search and clustering orders of magnitude faster than BLAST. *Bioinformatics* 26 2460–2461. 10.1093/bioinformatics/btq461 20709691

[B37] Eisen J. A. Heidelberg J. F. White O. Salzberg S. L. (2000). Evidence for symmetric chromosomal inversions around the replication origin in bacteria. *Genome Biol.* 1:RESEARCH0011. 10.1186/gb-2000-1-6-research0011 11178265 PMC16139

[B38] Elbourne L. D. Tetu S. G. Hassan K. A. Paulsen I. T. (2017). TransportDB 2.0: a database for exploring membrane transporters in sequenced genomes from all domains of life. *Nucleic Acids Res.* 45 D320–D324. 10.1093/nar/gkw1068 27899676 PMC5210551

[B39] Eng C. Thibessard A. Danielsen M. Rasmussen T. B. Mari J. F. Leblond P. (2011). *In silico* prediction of horizontal gene transfer in *Streptococcus thermophilus*. *Arch. Microbiol.* 193 287–297. 10.1007/s00203-010-0671-8 21234750

[B40] Ercolini D. Fusco V. Blaiotta G. Coppola S. (2005). Sequence heterogeneity in the lacSZ operon of *Streptococcus thermophilus* and its use in PCR systems for strain differentiation. *Res. Microbiol.* 156 161–172. 10.1016/j.resmic.2004.09.005 15748980

[B41] European Food Safety Authority [EFSA], (2007). Opinion of the scientific committee on a request from EFSA on the introduction of a Qualified Presumption of Safety (QPS) approach for assessment of selected microorganisms referred to EFSA. *EFSA J.* 587 1–16. 10.2903/j.efsa.2007.587

[B42] Evivie S. E. Li B. Ding X. Meng Y. Yu S. Du J. (2017). Complete genome sequence of *Streptococcus thermophilus* KLDS 3.1003, a strain with high antimicrobial potential against foodborne and vaginal pathogens. *Front. Microbiol.* 8:1238. 10.3389/fmicb.2017.01238 28744258 PMC5504653

[B43] Fernandez-Gomez B. Fernandez-Guerra A. Casamayor E. O. Gonzalez J. M. Pedros-Alio C. Acinas S. G. (2012). Patterns and architecture of genomic islands in marine bacteria. *BMC Genomics* 13:347. 10.1186/1471-2164-13-347 22839777 PMC3478194

[B44] Food and Drug Administration [FDA] (2007). *21 CFR Part 131: Microorganisms & Microbial-Derived Ingredients Used in Food (Partial List).* Silver Spring, MA: FDA.

[B45] Galia W. Jameh N. Perrin C. Genay M. Dary-Mourot A. (2016). Acquisition of PrtS in *Streptococcus thermophilus* is not enough in certain strains to achieve rapid milk acidification. *Dairy Sci. Technol.* 96 623–636. 10.1007/s13594-016-0292-3 19915034

[B46] Galia W. Perrin C. Genay M. Dary A. (2009). Variability and molecular typing of *Streptococcus thermophilus* strains displaying different proteolytic and acidifying properties. *Int. Dairy J.* 19 89–95. 10.1016/j.idairyj.2008.08.004

[B47] Garault P. Letort C. Juillard V. Monnet V. (2000). Branched-chain amino acid biosynthesis is essential for optimal growth of *Streptococcus thermophilus* in milk. *Appl. Environ. Microbiol.* 66 5128–5133. 10.1128/aem.66.12.5128-5133.2000 11097879 PMC92433

[B48] Gardini F. Rossi F. Rizzotti L. Torriani S. Grazia L. Chiavari C. (2012). Role of *Streptococcus thermophilus* PRI60 in histamine accumulation in cheese. *Int. Dairy J.* 27 71–76. 10.1016/j.idairyj.2012.07.005

[B49] Geertsma E. R. Duurkens R. H. Poolman B. (2005). The activity of the lactose transporter from *Streptococcus thermophilus* is increased by phosphorylated IIA and the action of β-galactosidase. *Biochemistry* 44 15889–15897. 10.1021/bi051638w 16313191

[B50] Giaretta S. Treu L. Vendramin V. Da Silva Duarte V. Tarrah A. Campanaro S. (2018). Comparative transcriptomic analysis of *Streptococcus thermophilus* TH1436 and TH1477 showing different capability in the use of galactose. *Front. Microbiol.* 9:1765. 10.3389/fmicb.2018.01765 30131781 PMC6090898

[B51] Giraffa G. Paris A. Valcavi L. Gatti M. Neviani E. (2001). Genotypic and phenotypic heterogeneity of *Streptococcus thermophilus* strains isolated from dairy products. *J. Appl. Microbiol.* 91 937–943. 10.1046/j.1365-2672.2001.01464.x 11722674

[B52] Goh Y. J. Goin C. O’flaherty S. Altermann E. Hutkins R. (2011). Specialized adaptation of a lactic acid bacterium to the milk environment: the comparative genomics of *Streptococcus thermophilus* LMD-9. *Microb. Cell Fact.* 10(Suppl. 1):S22. 10.1186/1475-2859-10-S1-S22 21995282 PMC3231929

[B53] Goris J. Konstantinidis K. T. Klappenbach J. A. Coenye T. Vandamme P. Tiedje J. M. (2007). DNA–DNA hybridization values and their relationship to whole-genome sequence similarities. *Int. J. Syst. Evol. Microbiol.* 57 81–91. 10.1099/ijs.0.64483-0 17220447

[B54] Grissa I. Vergnaud G. Pourcel C. (2007). CRISPRFinder: a web tool to identify clustered regularly interspaced short palindromic repeats. *Nucleic Acids Res.* 35 W52–W57. 10.1093/nar/gkm360 17537822 PMC1933234

[B55] Hafeez Z. Cakir-Kiefer C. Girardet J.-M. Lecomte X. Paris C. Galia W. (2015). New insights into the proteolytic system of *Streptococcus thermophilus*: use of isracidin to characterize cell-associated extracellular peptidase activities. *J. Agric. Food Chem.* 63 7522–7531. 10.1021/acs.jafc.5b01647 26193375

[B56] Hafeez Z. Cakir-Kiefer C. Lecomte X. Miclo L. Dary-Mourot A. (2019). The X-prolyl dipeptidyl-peptidase PepX of *Streptococcus thermophilus* initially described as intracellular is also responsible for peptidase extracellular activity. *J. Dairy Sci.* 102 113–123. 10.3168/jds.2018-14823 30391182

[B57] Han X. Yang Z. Jing X. Yu P. Zhang Y. Yi H. (2016). Improvement of the texture of yogurt by use of exopolysaccharide producing lactic acid bacteria. *Biomed. Res. Int.* 2016:7945675. 10.1155/2016/7945675 27294135 PMC4884582

[B58] Hao M. Cui Y. Qu X. (2018). Analysis of CRISPR-Cas system in *Streptococcus thermophilus* and its application. *Front. Microbiol.* 9:257. 10.3389/fmicb.2018.00257 29515542 PMC5826314

[B59] Hatmaker E. A. Riley L. A. O’dell K. B. Papanek B. Graveley B. R. Garrett S. C. (2018). Complete genome sequence of industrial dairy strain *Streptococcus thermophilus* DGCC 7710. *Genome Announc.* 6:e01587-17. 10.1128/genomeA.01587-17 29439051 PMC5805889

[B60] Herve-Jimenez L. Guillouard I. Guedon E. Boudebbouze S. Hols P. Monnet V. (2009). Postgenomic analysis of *Streptococcus thermophilus* cocultivated in milk with *Lactobacillus delbrueckii* subsp. *bulgaricus*: involvement of nitrogen, purine, and iron metabolism. *Appl. Environ. Microbiol.* 75 2062–2073. 10.1128/AEM.01984-08 19114510 PMC2663229

[B61] Hols P. Hancy F. Fontaine L. Grossiord B. Prozzi D. Leblond-Bourget N. (2005). New insights in the molecular biology and physiology of *Streptococcus thermophilus* revealed by comparative genomics. *FEMS Microbiol. Rev.* 29 435–463. 10.1016/j.fmrre.2005.04.008 16125007

[B62] Horvath P. Barrangou R. (2010). CRISPR/Cas, the immune system of bacteria and archaea. *Science* 327 167–170. 10.1126/science.1179555 20056882

[B63] Horvath P. Romero D. A. Coute-Monvoisin A. C. Richards M. Deveau H. Moineau S. (2008). Diversity, activity, and evolution of CRISPR loci in *Streptococcus thermophilus*. *J. Bacteriol.* 190 1401–1412. 10.1128/JB.01415-07 18065539 PMC2238196

[B64] Huang Y. Niu B. Gao Y. Fu L. Li W. (2010). CD-HIT Suite: a web server for clustering and comparing biological sequences. *Bioinformatics* 26 680–682. 10.1093/bioinformatics/btq003 20053844 PMC2828112

[B65] Huerta-Cepas J. Forslund K. Coelho L. P. Szklarczyk D. Jensen L. J. Von Mering C. (2017). Fast genome-wide functional annotation through orthology assignment by eggNOG-mapper. *Mol. Biol. Evol.* 34 2115–2122. 10.1093/molbev/msx148 28460117 PMC5850834

[B66] Huerta-Cepas J. Szklarczyk D. Forslund K. Cook H. Heller D. Walter M. C. (2016). eggNOG 4.5: a hierarchical orthology framework with improved functional annotations for eukaryotic, prokaryotic and viral sequences. *Nucleic Acids Res.* 44 D286–D293. 10.1093/nar/gkv1248 26582926 PMC4702882

[B67] Iyer R. Tomar S. K. Uma Maheswari T. Singh R. (2010). *Streptococcus thermophilus* strains: multifunctional lactic acid bacteria. *Int. Dairy J.* 20 133–141. 10.1016/j.idairyj.2009.10.005

[B68] Jans C. Follador R. Hochstrasser M. Lacroix C. Meile L. Stevens M. J. A. (2013a). Comparative genome analysis of *Streptococcus infantarius* subsp. *infantarius* CJ18, an African fermented camel milk isolate with adaptations to dairy environment. *BMC Genomics* 14:200. 10.1186/1471-2164-14-200 23521820 PMC3640971

[B69] Jans C. Kaindi D. W. M. Böck D. Njage P. M. K. Kouamé-Sina S. M. Bonfoh B. (2013b). Prevalence and comparison of *Streptococcus infantarius* subsp. *infantarius* and *Streptococcus gallolyticus* subsp. *macedonicus* in raw and fermented dairy products from East and West Africa. *Int. J. Food Microbiol.* 167 186–195. 10.1016/j.ijfoodmicro.2013.09.008 24131584 PMC4881808

[B70] Juhas M. Van Der Meer J. R. Gaillard M. Harding R. M. Hood D. W. Crook D. W. (2009). Genomic islands: tools of bacterial horizontal gene transfer and evolution. *FEMS Microbiol. Rev.* 33 376–393. 10.1111/j.1574-6976.2008.00136.x 19178566 PMC2704930

[B71] Junges R. Maienschein-Cline M. Morrison D. A. Petersen F. C. (2019). Complete genome sequence of *Streptococcus pneumoniae* serotype 19F strain EF3030. *Microbiol. Resour. Announc.* 8:e00198-19. 10.1128/MRA.00198-19 31072896 PMC6509521

[B72] Kanehisa M. Sato Y. Kawashima M. Furumichi M. Tanabe M. (2016a). KEGG as a reference resource for gene and protein annotation. *Nucleic Acids Res.* 44 D457–D462. 10.1093/nar/gkv1070 26476454 PMC4702792

[B73] Kanehisa M. Sato Y. Morishima K. (2016b). BlastKOALA and GhostKOALA: KEGG tools for functional characterization of genome and metagenome sequences. *J. Mol. Biol.* 428 726–731. 10.1016/j.jmb.2015.11.006 26585406

[B74] Kang X. Ling N. Sun G. Zhou Q. Zhang L. Sheng Q. (2012). Complete genome sequence of *Streptococcus thermophilus* strain MN-ZLW-002. *J. Bacteriol.* 194 4428–4429. 10.1128/JB.00740-12 22843572 PMC3416250

[B75] Kelleher P. Bottacini F. Mahony J. Kilcawley K. N. Van Sinderen D. (2017). Comparative and functional genomics of the *Lactococcus lactis* Taxon; insights into evolution and niche adaptation. *BMC Genomics* 18:267. 10.1186/s12864-017-3650-5 28356072 PMC5372332

[B76] Kongo J. M. (2013). “Lactic acid bacteria as starter-cultures for cheese processing: past, present and future developments,” in *Lactic Acid Bacteria - R & D for Food, Health and Livestock Purposes*, ed. Kongo J. M., (London: IntechOpen), 10.5772/55937

[B77] Labrie S. J. Tremblay D. M. Plante P. L. Wasserscheid J. Dewar K. Corbeil J. (2015). Complete genome sequence of *Streptococcus thermophilus* SMQ-301, a model strain for phage-host interactions. *Genome Announc.* 3:e00480-15. 10.1128/genomeA.00480-15 25999573 PMC4440953

[B78] Lefébure T. Stanhope M. J. (2007). Evolution of the core and pan-genome of *Streptococcus*: positive selection, recombination, and genome composition. *Genome Biol.* 8:R71. 10.1186/gb-2007-8-5-r71 17475002 PMC1929146

[B79] Lerat L. Ochman H. (2005). Recognizing the pseudogenes in bacterial genomes. *Nucleic Acids Res.* 33 3125–3132. 10.1093/nar/gki631 15933207 PMC1142405

[B80] Letort C. Juillard V. (2001). Development of a minimal chemically-defined medium for the exponential growth of *Streptococcus thermophilus*. *J. Appl. Microbiol.* 91 1023–1029. 10.1046/j.1365-2672.2001.01469.x 11851809

[B81] Li B. Ding X. Evivie S. E. Jin D. Meng Y. Huo G. (2018). Short communication: genomic and phenotypic analyses of exopolysaccharides produced by *Streptococcus thermophilus* KLDS SM. *J. Dairy Sci.* 101 106–112. 10.3168/jds.2017-13534 29055533

[B82] Limauro D. Falciatore A. Basso A. L. Forlani G. De Felice M. (1996). Proline biosynthesis in *Streptococcus thermophilus*: characterization of the proBA operon and its products. *Microbiology* 142 3275–3282. 10.1099/13500872-142-11-3275 8969524

[B83] Linares D. M. Arboleya S. Ross R. P. Stanton C. (2017). Complete genome sequence of the gamma-aminobutyric acid-producing strain *Streptococcus thermophilus* APC151. *Genome Announc.* 5:e00205-17. 10.1128/genomeA.00205-17 28450504 PMC5408102

[B84] Linares D. M. O’callaghan T. F. O’connor P. M. Ross R. P. Stanton C. (2016). *Streptococcus thermophilus* APC151 strain is suitable for the manufacture of naturally GABA-enriched bioactive yogurt. *Front. Microbiol.* 7:1876. 10.3389/fmicb.2016.01876 27920772 PMC5118970

[B85] Liu M. Bayjanov J. R. Renckens B. Nauta A. Siezen R. J. (2010). The proteolytic system of lactic acid bacteria revisited: a genomic comparison. *BMC Genomics* 11:36. 10.1186/1471-2164-11-36 20078865 PMC2827410

[B86] Liu M. Siezen R. J. Nauta A. (2009). *In silico* prediction of horizontal gene transfer events in *Lactobacillus bulgaricus* and *Streptococcus thermophilus* reveals protocooperation in yogurt manufacturing. *Appl. Environ. Microbiol.* 75 4120–4129. 10.1128/AEM.02898-08 19395564 PMC2698337

[B87] Lluis-Arroyo D. Flores-Nájera A. Cruz-Guerrero A. Gallardo-Escamilla F. Lobato-Calleros C. Jiménez-Guzmán J. (2014). Effect of an exopolysaccharide-producing strain of *Streptococcus thermophilus* on the yield and texture of Mexican Manchego-type cheese. *Int. J. Food Prop.* 17 1680–1693. 10.1080/10942912.2011.599091

[B88] Louis E. P. Wei Y. Terns M. P. (2017). Investigating the molecular mechanism of CRISPR-Cas adaptation of *Streptococcus thermophilus*. *J. Immunol.* 198(Suppl. 1):67.11.

[B89] Makarova K. Slesarev A. Wolf Y. Sorokin A. Mirkin B. Koonin E. (2006). Comparative genomics of the lactic acid bacteria. *Proc. Natl. Acad. Sci. U.S.A.* 103 15611–15616. 10.1073/pnas.0607117103 17030793 PMC1622870

[B90] Makarova K. S. Wolf Y. I. Alkhnbashi O. S. Costa F. Shah S. A. Saunders S. J. (2015). An updated evolutionary classification of CRISPR-Cas systems. *Nat. Rev. Microbiol.* 13 722–736. 10.1038/nrmicro3569 26411297 PMC5426118

[B91] Manat G. Roure S. Auger R. Bouhss A. Barreteau H. Mengin-Lecreulx D. (2014). Deciphering the metabolism of undecaprenyl-phosphate: the bacterial cell-wall unit carrier at the membrane frontier. *Microb. Drug Resist.* 20 199–214. 10.1089/mdr.2014.0035 24799078 PMC4050452

[B92] Mayo B. Van Sinderen D. Ventura M. (2008). Genome analysis of food grade lactic Acid-producing bacteria: from basics to applications. *Curr. Genomics* 9 169–183. 10.2174/138920208784340731 19440514 PMC2679651

[B93] Monnet C. Mora D. Corrieu G. (2005). Glutamine synthesis is essential for growth of *Streptococcus thermophilus* in milk and is linked to urea catabolism. *Appl. Environ. Microbiol.* 71 3376–3378. 10.1128/AEM.71.6.3376-3378.2005 15933046 PMC1151851

[B94] Mora D. Fortina M. G. Parini C. Ricci G. Gatti M. Giraffa G. (2002). Genetic diversity and technological properties of *Streptococcus thermophilus* strains isolated from dairy products. *J. Appl. Microbiol.* 93 278–287. 10.1046/j.1365-2672.2002.01696.x 12147076

[B95] Mora D. Maguin E. Masiero M. Parini C. Ricci G. Manachini P. L. (2004). Characterization of urease genes cluster of *Streptococcus thermophilus*. *J. Appl. Microbiol.* 96 209–219. 10.1046/j.1365-2672.2003.02148.x 14678176

[B96] Mora D. Monnet C. Parini C. Guglielmetti S. Mariani A. Pintus P. (2005). Urease biogenesis in *Streptococcus thermophilus*. *Res. Microbiol.* 156 897–903. 10.1016/j.resmic.2005.04.005 16024230

[B97] Moschetti G. Blaiotta G. Aponte M. Catzeddu P. Villani F. Deiana P. (1998). Random amplified polymorphic DNA and amplified ribosomal DNA spacer polymorphism: powerful methods to differentiate *Streptococcus thermophilus* strains. *J. Appl. Microbiol.* 85 25–36. 10.1046/j.1365-2672.1998.00461.x 9721653

[B98] Ninova-Nikolova N. Urshev Z. (2013). Fast acidifying urease-deficient *Streptococcus thermophilus* isolate shows spontaneous deletion of its complete urease operon. *Bulg. J. Agric. Sci.* 19 112–116.

[B99] Nishimura J. Kawai Y. Aritomo R. Ito Y. Makino S. Ikegami S. (2013). Effect of formic acid on exopolysaccharide production in skim milk fermentation by *Lactobacillus delbrueckii* subsp. *bulgaricus* OLL1073R-1. *Biosci. Microbiota Food Health* 32 23–32. 10.12938/bmfh.32.23 24936359 PMC4034293

[B100] Padmanabhan A. Tong Y. Wu Q. Zhang J. Shah N. P. (2018). Transcriptomic insights into the growth phase- and sugar-associated changes in the exopolysaccharide production of a high EPS-producing *Streptococcus thermophilus* ASCC 1275. *Front. Microbiol.* 9:1919. 10.3389/fmicb.2018.01919 30177921 PMC6109772

[B101] Papadimitriou K. Anastasiou R. Maistrou E. Plakas T. Papandreou N. C. Hamodrakas S. J. (2015a). Acquisition through horizontal gene transfer of plasmid pSMA198 by *Streptococcus macedonicus* ACA-DC 198 points towards the dairy origin of the species. *PLoS One* 10:e0116337. 10.1371/journal.pone.0116337 25584532 PMC4293149

[B102] Papadimitriou K. Pot B. Tsakalidou E. (2015b). How microbes adapt to a diversity of food niches. *Curr. Opin. Food Sci.* 2 29–35. 10.1016/j.cofs.2015.01.001 23339658

[B103] Papadimitriou K. Anastasiou R. Mavrogonatou E. Blom J. Papandreou N. C. Hamodrakas S. J. (2014). Comparative genomics of the dairy isolate *Streptococcus macedonicus* ACA-DC 198 against related members of the *Streptococcus bovis*/*Streptococcus equinus* complex. *BMC Genomics* 15:272. 10.1186/1471-2164-15-272 24713045 PMC4051162

[B104] Pastink M. I. Teusink B. Hols P. Visser S. De Vos W. M. Hugenholtz J. (2009). Genome-scale model of *Streptococcus thermophilus* LMG18311 for metabolic comparison of lactic acid bacteria. *Appl. Environ. Microbiol.* 75 3627–3633. 10.1128/AEM.00138-09 19346354 PMC2687286

[B105] Pernoud S. Fremaux C. Sepulchre A. Corrieu G. Monnet C. (2004). Effect of the metabolism of urea on the acidifying activity of *Streptococcus thermophilus*. *J. Dairy Sci.* 87 550–555. 10.3168/jds.S0022-0302(04)73196-3 15202638

[B106] Proust L. Loux V. Martin V. Magnabosco C. Pedersen M. Monnet V. (2018). Complete genome sequence of the industrial fast-acidifying strain *Streptococcus thermophilus* N4L. *Microbiol. Resour. Announc.* 7:e01029-18. 10.1128/MRA.01029-18 30533920 PMC6256512

[B107] Purwandari U. Shah N. P. Vasiljevic T. (2007). Effects of exopolysaccharide-producing strains of *Streptococcus thermophilus* on technological and rheological properties of set-type yoghurt. *Int. Dairy J.* 17 1344–1352. 10.1016/j.idairyj.2007.01.018

[B108] Qiao Y. Liu G. Leng C. Zhang Y. Lv X. Chen H. (2018). Metabolic profiles of cysteine, methionine, glutamate, glutamine, arginine, aspartate, asparagine, alanine and glutathione in *Streptococcus thermophilus* during pH-controlled batch fermentations. *Sci. Rep.* 8:12441. 10.1038/s41598-018-30272-5 30127376 PMC6102215

[B109] Rantsiou K. Urso R. Dolci P. Comi G. Cocolin L. (2008). Microflora of Feta cheese from four Greek manufacturers. *Int. J. Food Microbiol.* 126 36–42. 10.1016/j.ijfoodmicro.2008.04.031 18555549

[B110] Renye J. A. Jr. Needleman D. S. Somkuti G. A. Steinberg D. H. (2017). Complete genome sequence of *Streptococcus thermophilus* strain B59671, which naturally produces the broad-spectrum bacteriocin thermophilin 110. *Genome Announc.* 5:e01213-17. 10.1128/genomeA.01213-17 29122869 PMC5679802

[B111] Renye J. A. Jr. Needleman D. S. Steinberg D. H. (2019). Complete genome sequences of bacteriocin-producing *Streptococcus thermophilus* strains ST106 and ST109. *Microbiol. Resour. Announc.* 8:e01336-18. 10.1128/MRA.01336-18 30801058 PMC6376417

[B112] Repar J. Warnecke T. (2017). Non-random inversion landscapes in prokaryotic genomes are shaped by heterogeneous selection pressures. *Mol. Biol. Evol.* 34 1902–1911. 10.1093/molbev/msx127 28407093 PMC5850607

[B113] Richter M. Rossello-Mora R. (2009). Shifting the genomic gold standard for the prokaryotic species definition. *Proc. Natl. Acad. Sci. U.S.A.* 106 19126–19131. 10.1073/pnas.0906412106 19855009 PMC2776425

[B114] Roberts R. J. Vincze T. Posfai J. Macelis D. (2005). REBASE–restriction enzymes and DNA methyltransferases. *Nucleic Acids Res.* 33 D230–D232. 10.1093/nar/gki029 15608184 PMC539983

[B115] Roberts R. J. Vincze T. Posfai J. Macelis D. (2015). REBASE–a database for DNA restriction and modification: enzymes, genes and genomes. *Nucleic Acids Res.* 43 D298–D299. 10.1093/nar/gku1046 25378308 PMC4383893

[B116] Rodriguez-R L. M. Gunturu S. Harvey W. T. Rosselló-Mora R. Tiedje J. M. Cole J. R. (2018). The Microbial Genomes Atlas (MiGA) webserver: taxonomic and gene diversity analysis of *Archaea* and *Bacteria* at the whole genome level. *Nucleic Acids Res.* 46 W282–W288. 10.1093/nar/gky467 29905870 PMC6031002

[B117] Sapranauskas R. Gasiunas G. Fremaux C. Barrangou R. Horvath P. Siksnys V. (2011). The *Streptococcus thermophilus* CRISPR/Cas system provides immunity in *Escherichia coli*. *Nucleic Acids Res.* 39 9275–9282. 10.1093/nar/gkr606 21813460 PMC3241640

[B118] Savijoki K. Ingmer H. Varmanen P. (2006). Proteolytic systems of lactic acid bacteria. *Appl. Microbiol. Biotechnol.* 71 394–406. 10.1007/s00253-006-0427-1 16628446

[B119] Scott E. J. II Luke-Marshall N. R. Campagnari A. A. Dyer D. W. (2019). Draft genome sequence of pediatric otitis media isolate *Streptococcus pneumoniae* strain EF3030, which forms *in vitro* biofilms that closely mimic *in vivo* biofilms. *Microbiol. Resour. Announc.* 8:e01114-18. 10.1128/MRA.01114-18 30643873 PMC6328646

[B120] Selle K. Klaenhammer T. R. Barrangou R. (2015). CRISPR-based screening of genomic island excision events in bacteria. *Proc. Natl. Acad. Sci. U.S.A.* 112 8076–8081. 10.1073/pnas.1508525112 26080436 PMC4491743

[B121] Settachaimongkon S. Nout M. J. Antunes Fernandes E. C. Hettinga K. A. Vervoort J. M. Van Hooijdonk T. C. (2014). Influence of different proteolytic strains of *Streptococcus thermophilus* in co-culture with *Lactobacillus delbrueckii* subsp. *bulgaricus* on the metabolite profile of set-yoghurt. *Int. J. Food Microbiol.* 177 29–36. 10.1016/j.ijfoodmicro.2014.02.008 24598513

[B122] Shi Y. Chen Y. Li Z. Yang L. Chen W. Mu Z. (2015). Complete genome sequence of *Streptococcus thermophilus* MN-BM-A02, a rare strain with a high acid-producing rate and low post-acidification ability. *Genome Announc.* 3:e00979-15. 10.1128/genomeA.00979-15 26337884 PMC4559733

[B123] Sieuwerts S. Molenaar D. Van Hijum S. A. Beerthuyzen M. Stevens M. J. Janssen P. W. (2010). Mixed-culture transcriptome analysis reveals the molecular basis of mixed-culture growth in *Streptococcus thermophilus* and *Lactobacillus bulgaricus*. *Appl. Environ. Microbiol.* 76 7775–7784. 10.1128/AEM.01122-10 20889781 PMC2988612

[B124] Solovyev V. Salamov A. (2011). “Automatic annotation of microbial genomes and metagenomic sequences,” in *Metagenomics and its Applications in Agriculture, Biomedicine and Environmental Studies*, ed. Li R. W., (New York, NY: Nova Science Publishers), 61–78.

[B125] Song X. Huang H. Xiong Z. Xia Y. Wang G. Yin B. (2018). Characterization of a cryptic plasmid isolated from *Lactobacillus casei* CP002616 and construction of shuttle vectors based on its Replicon. *J. Dairy Sci.* 101 2875–2886. 10.3168/jds.2017-13771 29428762

[B126] Sørensen K. I. Curic-Bawden M. Junge M. P. Janzen T. Johansen E. (2016). Enhancing the sweetness of yoghurt through metabolic remodeling of carbohydrate metabolism in *Streptococcus thermophilus* and *Lactobacillus delbrueckii* subsp. *bulgaricus*. *Appl. Environ. Microbiol.* 82 3683–3692. 10.1128/AEM.00462-16 27107115 PMC4959173

[B127] Sujoy B. Aparna A. (2013). Enzymology, immobilization and applications of urease enzyme. *Int. Res. J. Biol. Sci.* 2 51–56.

[B128] Sullivan M. J. Petty N. K. Beatson S. A. (2011). Easyfig: a genome comparison visualizer. *Bioinformatics* 27 1009–1010. 10.1093/bioinformatics/btr039 21278367 PMC3065679

[B129] Sun Z. Chen X. Wang J. Zhao W. Shao Y. Wu L. (2011). Complete genome sequence of *Streptococcus thermophilus* strain ND03. *J. Bacteriol.* 193 793–794. 10.1128/JB.01374-10 21131489 PMC3021231

[B130] Tamulaitis G. Kazlauskienë M. Manakova E. Venclovas Č. Nwokeoji A. Dickman J. (2014). Programmable RNA shredding by the type III-A CRISPR-Cas system of *Streptococcus thermophilus*. *Mol. Cell* 56 506–517. 10.1016/j.molcel.2014.09.027 25458845

[B131] Tian H. Li B. Evivie S. E. Sarker S. K. Chowdhury S. Lu J. (2018). Technological and genomic analysis of roles of the cell-envelope protease PrtS in yoghurt starter development. *Int. J. Mol. Sci.* 19:E1068. 10.3390/ijms19041068 29614042 PMC5979370

[B132] Vaillancourt K. Bedard N. Bart C. Tessier M. Robitaille G. Turgeon N. (2008). Role of galK and galM in galactose metabolism by *Streptococcus thermophilus*. *Appl. Environ. Microbiol.* 74 1264–1267. 10.1128/AEM.01585-07 18065633 PMC2258605

[B133] Van Mastrigt O. Di Stefano E. Hartono S. Abee T. Smid E. J. (2018). Large plasmidome of dairy *Lactococcus lactis* subsp. *lactis* biovar diacetylactis FM03P encodes technological functions and appears highly unstable. *BMC Genomics* 19:620. 10.1186/s12864-018-5005-2 30119641 PMC6098607

[B134] Vaughan E. E. Kleerebezem M. de Vos W. M. (2003). “Genetics of the metabolism of lactose and other sugars,” in *Genetics of Lactic Acid Bacteria*, eds Wood B. J. B. Warner P. J., (New York, NY: Kluwer Academic), 95–119. 10.1007/978-1-4615-7090-5_4

[B135] Vaughan E. E. Van Den Bogaard P. T. Catzeddu P. Kuipers O. P. De Vos W. M. (2001). Activation of silent *gal* genes in the *lac-gal* regulon of *Streptococcus thermophilus*. *J. Bacteriol.* 183 1184–1194. 10.1128/JB.183.4.1184-1194.2001 11157930 PMC94991

[B136] Wang T. Xu Z. Lu S. Xin M. Kong J. (2016). Effects of glutathione on acid stress resistance and symbiosis between *Streptococcus thermophilus* and *Lactobacillus delbrueckii* subsp. *bulgaricus*. *Int. Dairy J.* 61 22–28. 10.1016/j.idairyj.2016.03.012

[B137] Wassenaar T. M. Lukjancenko O. (2014). “Comparative genomics of *Lactobacillus* and other LAB,” in *Lactic Acid Bacteria: Biodiversity and Taxonomy*, eds Holzapfel W. H. Wood B. J. B., (Hoboken, NJ: John Wiley & Sons), 55–69. 10.1002/9781118655252.ch5

[B138] Wu Q. Shah N. P. (2018). Comparative mRNA-Seq analysis reveals the improved EPS production machinery in *Streptococcus thermophilus* ASCC 1275 during optimized milk fermentation. *Front. Microbiol.* 9:445. 10.3389/fmicb.2018.00445 29593689 PMC5859087

[B139] Wu Q. Tun H. M. Leung F. C. Shah N. P. (2014). Genomic insights into high exopolysaccharide-producing dairy starter bacterium *Streptococcus thermophilus* ASCC 1275. *Sci. Rep.* 4:4974. 10.1038/srep04974 24827399 PMC4021336

[B140] Xiong Z.-Q. Kong L.-H. Lai P. F. H. Xia Y.-J. Liu J.-C. Li Q.-Y. (2019a). Genomic and phenotypic analyses of exopolysaccharide biosynthesis in *Streptococcus thermophilus* S-3. *J. Dairy Sci.* 102 4925–4934. 10.3168/jds.2018-15572 30928267

[B141] Xiong Z.-Q. Kong L.-H. Meng H.-L. Cui J.-M. Xia Y.-J. Wang S.-J. (2019b). Comparison of gal-lac operons in wild-type galactose-positive and -negative *Streptococcus thermophilus* by genomics and transcription analysis. *J. Ind. Microbiol. Biotechnol.* 46 751–758. 10.1007/s10295-019-02145-x 30715626

[B142] Yamauchi R. Maguin E. Horiuchi H. Hosokawa M. Sasaki Y. (2019). The critical role of urease in yogurt fermentation with various combinations of *Streptococcus thermophilus* and *Lactobacillus delbrueckii* ssp. *bulgaricus*. *J. Dairy Sci.* 102 1033–1043. 10.3168/jds.2018-15192 30594386

[B143] Yu J. Sun Z. Liu W. Xi X. Song Y. Xu H. (2015). Multilocus sequence typing of *Streptococcus thermophilus* from naturally fermented dairy foods in China and Mongolia. *BMC Microbiol.* 15:236. 10.1186/s12866-015-0551-0 26497818 PMC4620635

[B144] Zotta T. Ricciardi A. Ciocia F. Rossano R. Parente E. (2008). Diversity of stress responses in dairy thermophilic streptococci. *Int. J. Food Microbiol.* 124 34–42. 10.1016/j.ijfoodmicro.2008.02.024 18407366

