# Supplementary material for: Comparative Genomics of Streptococcus thermophilus Support Important Traits Concerning the Evolution, Biology and Technological Properties of the Species
Source: Front Microbiol. 2019 Dec 20;10:2916. doi: 10.3389/fmicb.2019.02916 (PMC6951406; doi:10.3389/fmicb.2019.02916)
Supplement: Supplementary file 1 [file Table_1.docx]

**Supplementary Table S1.** Number of accessory, unique and exclusively absent genes resulted from pan genome analysis of the 23 *S. thermophilus* strains presented according to the clusters and subgroups described in the text

| **Cluster** | **Subgroup** | **Strain** | **Genes in**  **accessory genome** | **Unique genes** | **Exclusively absent genes** |
| --- | --- | --- | --- | --- | --- |
| No cluster |  | NCTC12958^T^ | 501 | 204 | 31 |
| **A** | No subgroup | KLDS 3.1003 | 519 | 41 | 13 |
|  | **I** | ASCC 1275 | 532 | 0 | 4 |
|  |  | ND07 | 541 | 0 | 2 |
|  |  | DGCC 7710 | 526 | 3 | 0 |
|  |  | KLDS SM | 538 | 1 | 1 |
|  |  | MN-BM-A02 | 539 | 0 | 1 |
|  | **II** | MN-ZLW-002 | 564 | 1 | 0 |
|  |  | MN-BM-A01 | 522 | 0 | 28 |
|  | No subgroup | JIM 8232 | 556 | 67 | 6 |
|  | **III** | LMD-9 | 535 | 7 | 5 |
|  |  | SMQ-301 | 536 | 13 | 0 |
|  | **IV** | ND03 | 568 | 0 | 0 |
|  |  | APC151 | 562 | 0 | 4 |
|  | No subgroup | GABA | 485 | 17 | 3 |
|  | No subgroup | ST3 | 501 | 12 | 7 |
| **B** | **I** | CNRZ1066 | 525 | 0 | 3 |
|  |  | CS8 | 526 | 0 | 2 |
|  |  | S9 | 513 | 0 | 0 |
|  |  | EPS | 481 | 15 | 13 |
|  | No subgroup | LMG 18311 | 483 | 20 | 7 |
|  | No subgroup | B59671 | 433 | 22 | 29 |
|  | No subgroup | ACA-DC 2 | 432 | 14 | 10 |
