# Supplementary material for: Comparative Genomics of Streptococcus thermophilus Support Important Traits Concerning the Evolution, Biology and Technological Properties of the Species
Source: Front Microbiol. 2019 Dec 20;10:2916. doi: 10.3389/fmicb.2019.02916 (PMC6951406; doi:10.3389/fmicb.2019.02916)
Supplement: Supplementary file 4 [file Table_4.docx]

**Supplementary Table S4.** General characteristics of exopolysaccharide (EPS) clusters identified in the 23 *S. thermophilus* strains. Dashed lines are used to separate the two major clusters (A and B) and strain NCTC12958^T^ of the species, as described in the text

| **Strain** | **EPS coordinates** | **EPS length (bp)** | **Genes** | **CDS** | **Pseudogenes** | **Unique CDS** | **GC (%)** |
| --- | --- | --- | --- | --- | --- | --- | --- |
| NCTC12958^T^ | com(1149594..1171095) | 21,502 | 24 | 20 | 4 | 1 | 36.4 |
| KLDS 3.1003 | com(1605450..1627455) | 22,006 | 24 | 15 | 9 | 1 | 34,9 |
| ASCC 1275 | com(1167275..1190619) | 23,345 | 26 | 15 | 11 | 0 | 35,0 |
| ND07 | 1663576..1686919 | 23,344 | 25 | 15 | 10 | 0 | 35,0 |
| DGCC 7710 | com(980395..1003738) | 23,344 | 26 | 17 | 9 | 0 | 35,0 |
| KLDS SM | com(292240..315583) | 23,344 | 25 | 15 | 10 | 0 | 35,0 |
| MN-BM-A02 | com(977025..1000368) | 23,344 | 25 | 15 | 10 | 0 | 35,0 |
| MN-ZLW-002 | 792502..828474 | 35,973 | 41 | 32 | 9 | 1 | 36,0 |
| MN-BM-A01 | 606167..642133 | 35,967 | 42 | 32 | 10 | 0 | 36,0 |
| JIM 8232 | com(1049941..1078711) | 28,771 | 27 | 23 | 4 | 3 | 36,4 |
| LMD-9 | com(975018..996065) | 21,048 | 21 | 16 | 5 | 5 | 34,7 |
| SMQ-301 | com(977441..1005388) | 27,948 | 27 | 17 | 10 | 4 | 34,3 |
| ND03 | com(951312..977745) | 26,434 | 34 | 23 | 11 | 0 | 35,1 |
| APC151 | com(163250..189683) | 26,434 | 34 | 23 | 11 | 0 | 35,1 |
| GABA | com(979718..1002037) | 22,320 | 24 | 19 | 5 | 0 | 35.7 |
| ST3 | com(1000985..1025295) | 24,311 | 28 | 20 | 8 | 0 | 34,6 |
| CNRZ1066 | com(960253..981421) | 21,169 | 22 | 18 | 4 | 0 | 34,7 |
| CS8 | 1725876..1747044 | 21,169 | 22 | 17 | 5 | 0 | 34,7 |
| S9 | 1743015..1764183 | 21,169 | 22 | 17 | 5 | 0 | 34,7 |
| EPS | 783382..804549 | 21,168 | 23 | 18 | 5 | 0 | 34,7 |
| LMG 18311 | com(954247..983323) | 29,077 | 36 | 27 | 9 | 15 | 34,3 |
| B59671 | com(316701..347524) | 30,824 | 33 | 24 | 9 | 9 | 35,6 |
| ACA-DC 2 | com(928529..947189) | 18,661 | 21 | 20 | 1 | 6 | 34,8 |
