# Supplementary material for: Comparative Genomics of Streptococcus thermophilus Support Important Traits Concerning the Evolution, Biology and Technological Properties of the Species
Source: Front Microbiol. 2019 Dec 20;10:2916. doi: 10.3389/fmicb.2019.02916 (PMC6951406; doi:10.3389/fmicb.2019.02916)
Supplement: Supplementary file 8 [file Table_8.docx]

**Supplementary Table S8.** Characteristics of confirmed Clustered Regularly Interspaced Short Palindromic Repeats (CRISPR) arrays as predicted by CRISPRFinder in the 23 *S. thermophilus* strains. Dashed lines are used to separate the two major clusters (A and B) and strain NCTC12958^T^ of the species, as described in the text

| **Strain** | **CRISPR-Cas systems^1^** | **CRISPR array coordinates** | **Direct Repeat (DR) consensus** | **DR length** | **Spacers** |
| --- | --- | --- | --- | --- | --- |
| NCTC12958^T^ | CRISPR1 | 803250..805592 | GTTTTTGTACTCTCAAGATTTAAGTAACTGTACAAC | 36 | 35 |
|  | CRISPR3 | 1568082..1569571 | GTTTTGGAACCATTCGAAACAACACAGCTCTAAAAC | 36 | 22 |
| KLDS 3.1003 | CRISPR3 | 121258..122019 | GTTTTGGAACCATTCGAAACAACACAGCTCTAAAACT | 37 | 11 |
|  | CRISPR1 | 1254932..1255890 | GTTTTTGTACTCTCAAGATTTAAGTAACTGTACAAC | 36 | 14 |
|  | CRISPR2 | 1506836..1507597 | GATATAAACCTAATTACCTCGAGAGGGGACGGAAAC | 36 | 10 |
| ASCC 1275 | CRISPR1 | 823246..825392 | GTTTTTGTACTCTCAAGATTTAAGTAACTGTACAAC | 36 | 32 |
|  | CRISPR2 | 1074866..1075124 | GATATAAACCTAATTACCTCGAGAGGGGACGGAAAC | 36 | 3 |
|  | CRISPR4 | 1144778..1145540 | GGATCACCCCCGCGTGTGCGGGAAAAAC | 28 | 12 |
|  | CRISPR3 | 1558841..1559668 | GTTTTGGAACCATTCGAAACAACACAGCTCTAAAAC | 36 | 12 |
| ND07 | CRISPR1 | 177242..179388 | GTTGTACAGTTACTTAAATCTTGAGAGTACAAAAAC | 36 | 32 |
|  | CRISPR3 | 1294523..1295350 | GTTTTAGAGCTGTGTTGTTTCGAATGGTTCCAAAAC | 36 | 12 |
|  | CRISPR4 | 1708655..1709417 | GTTTTTCCCGCACACGCGGGGGTGATCC | 28 | 12 |
|  | CRISPR2 | 1779078..1779330 | GTCCCCTCTCGAGGTAATTAGGTTTATATC | 30 | 3 |
| DGCC 7710 | CRISPR1 | 636570..638716 | GTTTTTGTACTCTCAAGATTTAAGTAACTGTACAAC | 36 | 32 |
|  | CRISPR2 | 887984..888242 | GATATAAACCTAATTACCTCGAGAGGGGACGGAAAC | 36 | 3 |
|  | CRISPR4 | 957897..958659 | GGATCACCCCCGCGTGTGCGGGAAAAAC | 28 | 12 |
|  | CRISPR3 | 1371965..1372792 | GTTTTGGAACCATTCGAAACAACACAGCTCTAAAAC | 36 | 12 |
| KLDS SM | CRISPR2 | 199829..200087 | GATATAAACCTAATTACCTCGAGAGGGGACGGAAAC | 36 | 3 |
|  | CRISPR4 | 269742..270504 | GGATCACCCCCGCGTGTGCGGGAAAAAC | 28 | 12 |
|  | CRISPR3 | 683810..684637 | GTTTTGGAACCATTCGAAACAACACAGCTCTAAAAC | 36 | 12 |
|  | CRISPR1 | 1806995..1809141 | GTTTTTGTACTCTCAAGATTTAAGTAACTGTACAAC | 36 | 32 |
| MN-BM-A02 | CRISPR1 | 633203..635349 | GTTTTTGTACTCTCAAGATTTAAGTAACTGTACAAC | 36 | 32 |
|  | CRISPR2 | 884614..884872 | GATATAAACCTAATTACCTCGAGAGGGGACGGAAAC | 36 | 3 |
|  | CRISPR4 | 954527..955289 | GGATCACCCCCGCGTGTGCGGGAAAAAC | 28 | 12 |
|  | CRISPR3 | 1368586..1369413 | GTTTTGGAACCATTCGAAACAACACAGCTCTAAAAC | 36 | 12 |
| MN-ZLW-002 | CRISPR1 | 630165..632177 | GTTTTTGTACTCTCAAGATTTAAGTAACTGTACAAC | 36 | 30 |
|  | CRISPR3 | 1372720..1374472 | GTTTTGGAACCATTCGAAACAACACAGCTCTAAAAC | 36 | 26 |
| MN-BM-A01 | CRISPR1 | 443838..445850 | GTTTTTGTACTCTCAAGATTTAAGTAACTGTACAAC | 36 | 30 |
|  | CRISPR3 | 1186347..1188099 | GTTTTGGAACCATTCGAAACAACACAGCTCTAAAAC | 36 | 26 |
| JIM 8232 | CRISPR1 | 712332..715137 | GTTTTTGTACTCTCAAGATTTAAGTAACTGTACAAC | 36 | 42 |
|  | CRISPR2 | 969817..971087 | GATATAAACCTAATTACCTCGAGAGGGGACGGAAAC | 36 | 17 |
|  | orphan^2^ | 1452282..1452514 | GTTTTGGAACCATTCGAAACAACACAGCTCTAAAAC | 36 | 3 |
| LMD-9 | CRISPR1 | 649125..650217 | GTTTTTGTACTCTCAAGATTTAAGTAACTGTACAAC | 36 | 16 |
|  | CRISPR2 | 897070..897328 | GATATAAACCTAATTACCTCGAGAGGGGACGGAAAC | 36 | 3 |
|  | CRISPR3 | 1377229..1377794 | GTTTTGGAACCATTCGAAACAACACAGCTCTAAAAC | 36 | 8 |
| SMQ-301 | CRISPR1 | 649756..650847 | GTTTTTGTACTCTCAAGATTTAAGTAACTGTACAAC | 36 | 16 |
|  | CRISPR2 | 897843..898101 | GATATAAACCTAATTACCTCGAGAGGGGACGGAAAC | 36 | 3 |
|  | CRISPR3 | 1383838..1384867 | GTTTTGGAACCATTCGAAACAACACAGCTCTAAAAC | 36 | 15 |
| ND03 | CRISPR1 | 639512..641921 | GTTTTTGTACTCTCAAGATTTAAGTAACTGTACAAC | 36 | 36 |
|  | CRISPR3 | 1363057..1364415 | GTTTTGGAACCATTCGAAACAACACAGCTCTAAAAC | 36 | 20 |
| APC151 | CRISPR1 | 1689194..1691603 | GTTTTTGTACTCTCAAGATTTAAGTAACTGTACAAC | 36 | 36 |
|  | CRISPR3 | 575011..576303 | GTTTTGGAACCATTCGAAACAACACAGCTCTAAAAC | 36 | 19 |
| GABA | CRISPR1 | 652778..655449 | GTTTTTGTACTCTCAAGATTTAAGTAACTGTACAAC | 36 | 40 |
|  | CRISPR2 | 889434..889684 | GATATAAACCTAATTACCTCGAGAGGGGACGGAAAC | 36 | 3 |
|  | CRISPR3 | 1396122..1397147 | GTTTTGGAACCATTCGAAACAACACAGCTCTAAAAC | 36 | 15 |
| ST3 | CRISPR1 | 652775..653929 | GTTTTTGTACTCTCAAGATTTAAGTAACTGTACAAC | 36 | 17 |
|  | CRISPR2 | 914332..914582 | GATATAAACCTAATTACCTCGAGAGGGGACGGAAAC | 36 | 3 |
|  | CRISPR3 | 1399223..1400777 | GTTTTGGAACCATTCGAAACAACACAGCTCTAAAAC | 36 | 23 |
| LMG 18311 | CRISPR1 | 629901..632113 | GTTTTTGTACTCTCAAGATTTAAGTAACTGTACAAC | 36 | 33 |
|  | orphan^2^ | 774319..774515 | CATATCATGCATATTGTCCATAT | 23 | 4 |
|  | CRISPR2 | 863999..864323 | GATATAAACCTAATTACCTCGAGAGGGGACGGAAAC | 36 | 4 |
| CNRZ1066 | CRISPR1 | 625101..627844 | GTTTTTGTACTCTCAAGATTTAAGTAACTGTACAAC | 36 | 41 |
| CS8 | CRISPR1 | 287797..290540 | GTTGTACAGTTACTTAAATCTTGAGAGTACAAAAAC | 36 | 41 |
| S9 | CRISPR1 | 304100..305059 | GTTGTACAGTTACTTAAATCTTGAGAGTACAAAAAC | 36 | 14 |
| EPS | CRISPR1 | 1136592..1137352 | GTTGTACAGTTACTTAAATCTTGAGAGTACAAAAAC | 36 | 11 |
| B59671 | CRISPR1 | 1815698..1816920 | GTTTTTGTACTCTCAAGATTTAAGTAACTGTACAAC | 36 | 18 |
|  | CRISPR4 | 295226..295498 | GGATCACCCCCGCGTGTGCGGGAAAAAC | 28 | 4 |
| ACA-DC 2 | - | - | - | - | - |

**^1^**CRISPR systems are named as described by Horvath and Barrangou, 2010

**^2^**CRISPR array identified in the absence of Cas proteins
