# Supplementary material for: Comparative Genomics of Streptococcus thermophilus Support Important Traits Concerning the Evolution, Biology and Technological Properties of the Species
Source: Front Microbiol. 2019 Dec 20;10:2916. doi: 10.3389/fmicb.2019.02916 (PMC6951406; doi:10.3389/fmicb.2019.02916)
Supplement: Supplementary file 10 [file Table_10.docx]

**Supplementary Table S10.** Restriction-modification (R-M) systems predicted in the 23 *S. thermophilus* strains after manual curation. Dashed lines are used to separate the two major clusters (A and B) and strain NCTC12958^T^ of the species, as described in the text

|  | **Number of predicted R-M systems** | | | | |
| --- | --- | --- | --- | --- | --- |
| **Strain** | **Type I** | **Type II** | **Type III** | **Type IV** | **Total** |
| NCTC12958^T^ | 2 | 2 (2) | 1 | 0 | 5 |
| KLDS 3.1003 | 2 | 1 (1) | 0 | 1 | 4 |
| ASCC 1275 | 4 (3)^1^ | 0 | 1 (1) | 1 | 6 |
| ND07 | 3 (2) | 0 | 1 (1) | 1 | 5 |
| DGCC 7710 | 3 (2) | 0 | 0 | 1 | 4 |
| KLDS SM | 3 (2) | 0 | 1 (1) | 1 | 5 |
| MN-BM-A02 | 3 (2) | 0 | 1 (1) | 1 | 5 |
| MN-ZLW-002 | 3 (3) | 2 | 1 (1) | 0 | 6 |
| MN-BM-A01 | 2 (2) | 3 (1) | 0 | 0 | 5 |
| JIM 8232 | 4 (3) | 2 (2) | 1 (1) | 1 | 8 |
| LMD-9 | 3 (2) | 3 (1) | 0 | 0 | 6 |
| SMQ-301 | 2 (1) | 2 (1) | 0 | 0 | 4 |
| ND03 | 2 (1) | 3 (1) | 0 | 0 | 5 |
| APC151 | 1 | 3 (1) | 0 | 0 | 4 |
| GABA | 2 | 3 (2) | 1 | 0 | 6 |
| ST3 | 1 (1) | 1 | 0 | 1 | 3 |
| CNRZ1066 | 3 (2) | 3 (2) | 1 | 1 (1) | 8 |
| CS8 | 3 (2) | 2 (1) | 1 | 0 | 6 |
| S9 | 1 | 1 (1) | 1 | 0 | 3 |
| EPS | 2 | 1 (1) | 1 | 0 | 4 |
| LMG 18311 | 4 (3) | 2 (1) | 1 | 1 (1) | 8 |
| B59671 | 0 | 2 (1) | 1 | 0 | 3 |
| ACA-DC 2 | 1 | 1 (1) | 1 | 1 | 4 |

^1^Number in parentheses represent potentially inactivated R-M systems due to the presence of putative pseudogenes or the absence of one or more required protein subunits
