# Supplementary material for: Comparative Genomics of Streptococcus thermophilus Support Important Traits Concerning the Evolution, Biology and Technological Properties of the Species
Source: Front Microbiol. 2019 Dec 20;10:2916. doi: 10.3389/fmicb.2019.02916 (PMC6951406; doi:10.3389/fmicb.2019.02916)
Supplement: Supplementary file 11 [file Table_11.docx]

**Supplementary Table S11.** General characteristics of integrated genomic islands (GIs) identified in the 23 S. thermophilus strains. Dashed lines are used to separate the two major clusters (A and B) and strain NCTC12958^T^ of the species, as described in the text

| **Strain** | **Predicted GIs** | **Unique GIs** | **Size range (bp)** | **GC (%) range** | **CDSs / pseudogenes** | **GIs excluded**  **from analysis^1^** |
| --- | --- | --- | --- | --- | --- | --- |
| NCTC12958^T^ | 23 | 11 | 3,555-38,494 | 30,3-42,9 | 281/30 | GI 22 |
| KLDS 3.1003 | 12 | 1 | 4,378-22,858 | 26,5-39,1 | 111/37 | - |
| ASCC 1275 | 11 | 0 | 5,578-35,763 | 26,4-42,1 | 140/28 | GI 1 |
| ND07 | 12 | 0 | 4,070-38,272 | 26,1-42,3 | 144/28 | GI 7 |
| DGCC 7710 | 13 | 0 | 4,070-39,057 | 26,4-39,0 | 138/23 | GI 12 |
| KLDS SM | 13 | 1 | 4,070-38,272 | 26,4-42.1 | 152/19 | GI 7 |
| MN-BM-A02 | 13 | 0 | 4,070-39,601 | 26,4-42,1 | 156/22 | GI 12 |
| MN-ZLW-002 | 10 | 0 | 4,411-48,230 | 30,1-42,9 | 144/25 | - |
| MN-BM-A01 | 10 | 1 | 4,411-48,298 | 30,1-42,9 | 140/28 | - |
| JIM 8232 | 12 | 6 | 4,477-58,211 | 26,5-41,6 | 164/7 | GI 12 |
| LMD-9 | 10 | 1 | 4,426-39,145 | 26,4-40,9 | 140/13 | GI 10 |
| SMQ-301 | 9 | 0 | 4,426-38,272 | 26,4-39,5 | 128/13 | GI 9 |
| ND03 | 10 | 0 | 5,091-22,592 | 29,3-39,7 | 141/24 | GI 9 |
| APC151 | 10 | 0 | 5,091-22,592 | 29,4-39,8 | 136/24 | GI 6 |
| GABA | 12 | 4 | 3,555-39,209 | 26,4-45,2 | 137/16 | GI 12 |
| ST3 | 7 | 1 | 5,607-39,428 | 26,4-37,8 | 101/20 | GI 7 |
| CNRZ1066 | 11 | 0 | 4,087-25,706 | 29,9-39,7 | 118/31 | - |
| CS8 | 11 | 0 | 4,411-21,101 | 26,5-39,4 | 107/26 | - |
| S9 | 10 | 0 | 4,109-21,101 | 26,5-40,3 | 93/24 | - |
| EPS | 9 | 0 | 4,333-15,950 | 26,5-41,0 | 63/14 | - |
| LMG 18311 | 10 | 1 | 4,591-36,887 | 30,1-40,5 | 139/17 | GI 9 |
| B59671 | 5 | 2 | 4,173-10,973 | 30,2-40,6 | 35/8 | - |
| ACA-DC 2 | 10 | 2 | 4,150-14,221 | 30,1-38,9 | 72/18 | - |

The respective GIs include the array of ribosomal proteins and as false positive results were excluded from further analysis
