# Supplementary material for: Comparative Genomics of Streptococcus thermophilus Support Important Traits Concerning the Evolution, Biology and Technological Properties of the Species
Source: Front Microbiol. 2019 Dec 20;10:2916. doi: 10.3389/fmicb.2019.02916 (PMC6951406; doi:10.3389/fmicb.2019.02916)
Supplement: Supplementary file 14 [file Data_Sheet_1.PDF]

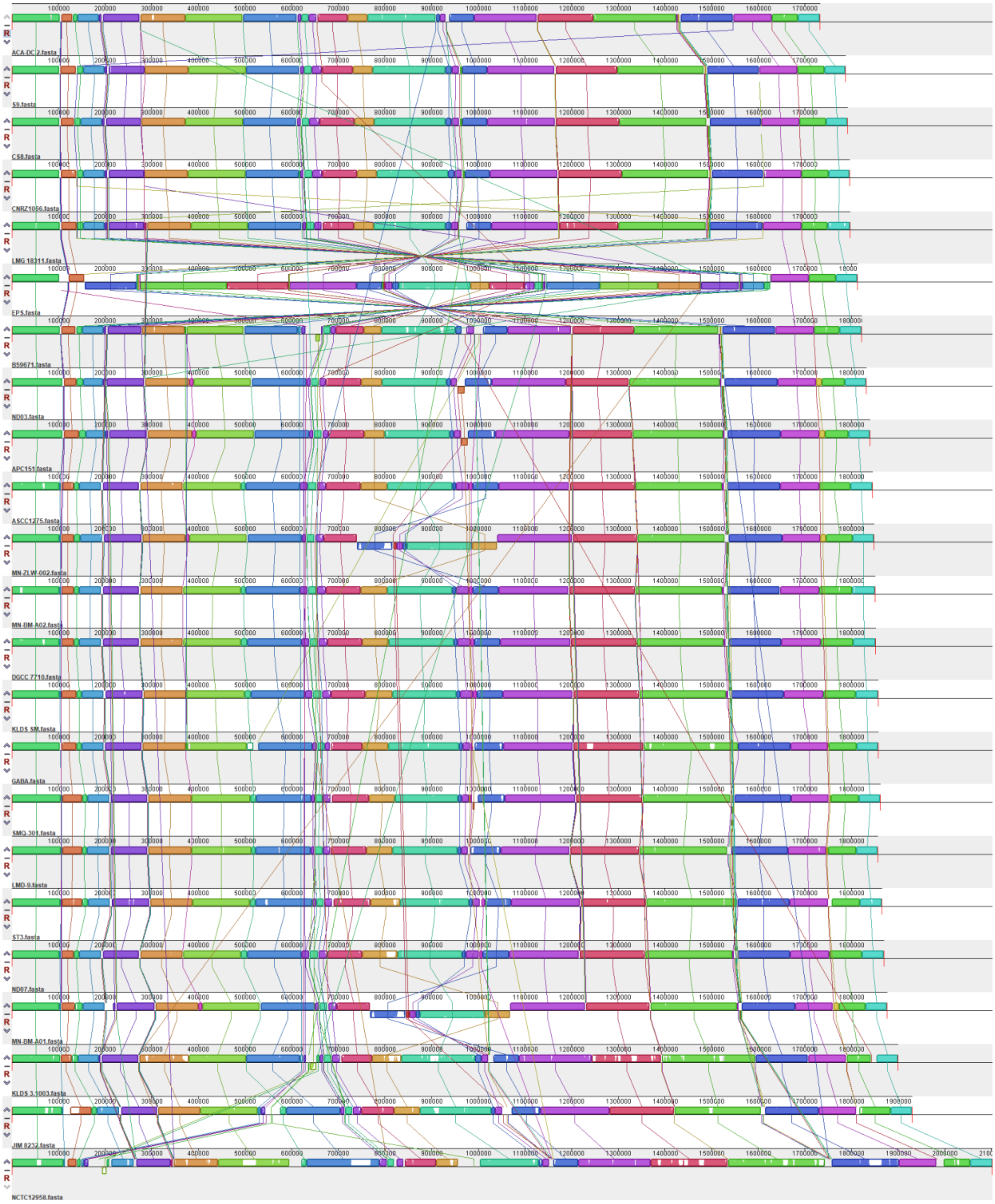

Supplementary Figure 1. Chromosome alignments of the 23 *S. thermophilus* strains with complete genomes as calculated by progressive Mauve. Local collinear blocks (LCBs) of conserved sequences among the strains are represented by rectangles of the same color. Connecting lines can be used to visualize synteny or rearrangement. LCBs positioned above or under the chromosome (blackline) correspond to the forward and reverse orientation, respectively. The level of conservation is equivalent to the level of vertical color filling within the LCBs (e.g. white regions are strain-specific). Sequences not placed within an LCB are unique for the particular strain. All genomes were synchronized from the *dnaA*
