# Supplementary material for: Comparative Genomics of Streptococcus thermophilus Support Important Traits Concerning the Evolution, Biology and Technological Properties of the Species
Source: Front Microbiol. 2019 Dec 20;10:2916. doi: 10.3389/fmicb.2019.02916 (PMC6951406; doi:10.3389/fmicb.2019.02916)
Supplement: Supplementary file 15 [file Data_Sheet_2.PDF]

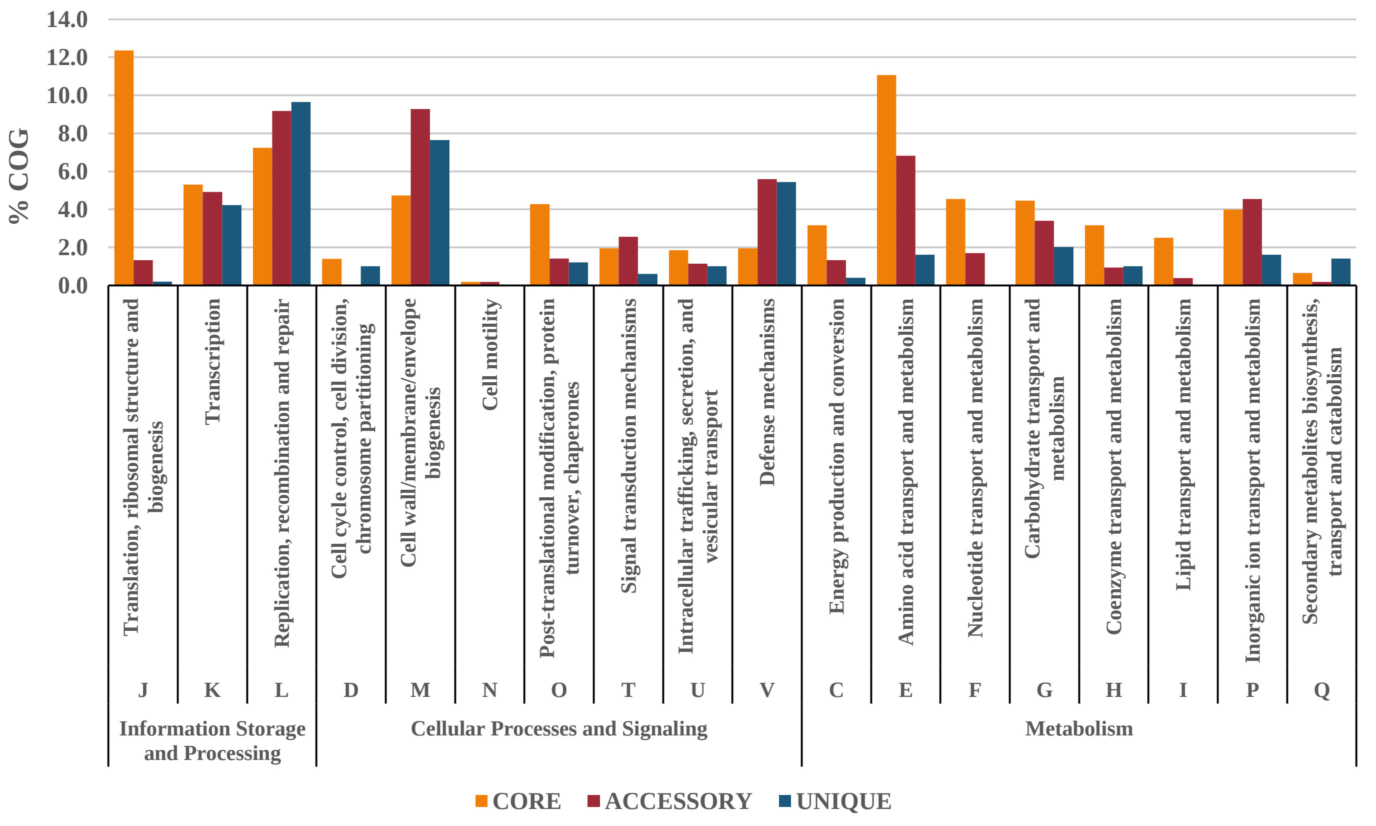

Supplementary Figure 2. Distribution of COG categories (%) for the core, accessory and unique genes identified in the 23 *S. thermophilus* strains
