# Supplementary material for: Comparative Genomics of Streptococcus thermophilus Support Important Traits Concerning the Evolution, Biology and Technological Properties of the Species
Source: Front Microbiol. 2019 Dec 20;10:2916. doi: 10.3389/fmicb.2019.02916 (PMC6951406; doi:10.3389/fmicb.2019.02916)
Supplement: Supplementary file 16 [file Data_Sheet_3.PDF]

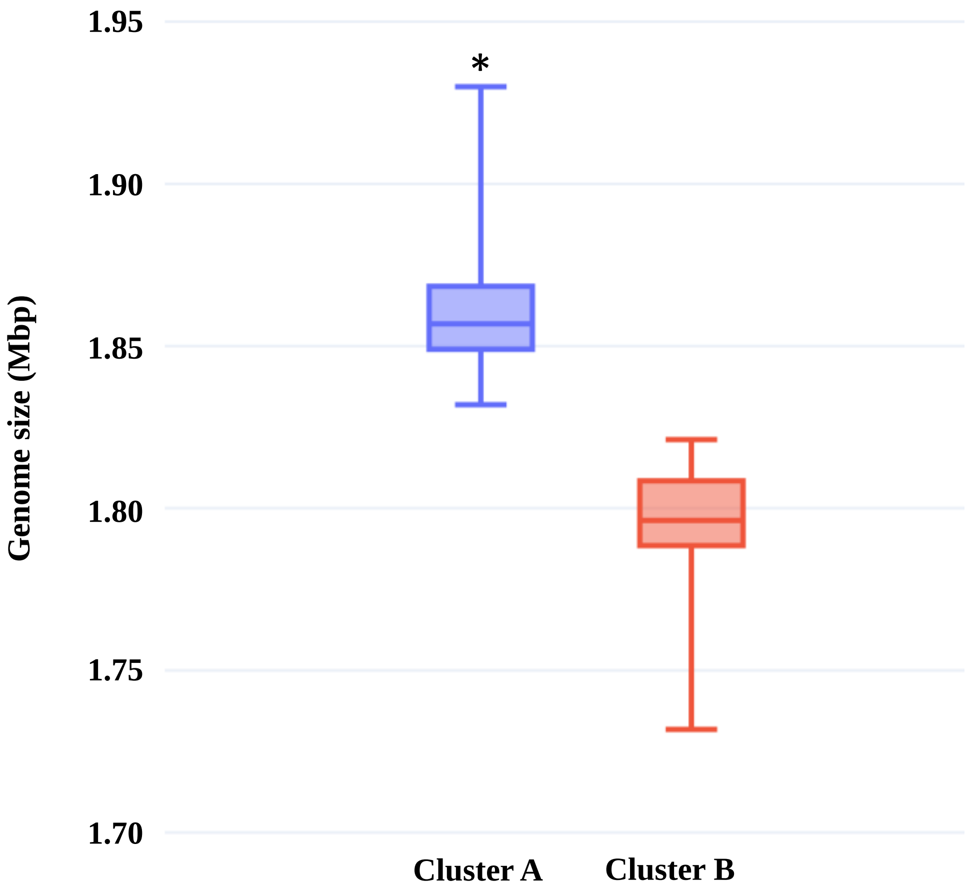

Supplementary Figure 3. Genome size distributions of cluster A and cluster B *S. thermophilus* strains analyzed in this study. The asterisk indicates significant difference between the two clusters as supported by the Mann-Whitney U Test ($p<0.05$).
