# Supplementary figures and images for: Comparative Genomics of Streptococcus thermophilus Support Important Traits Concerning the Evolution, Biology and Technological Properties of the Species
Source: Front Microbiol. 2019 Dec 20;10:2916. doi: 10.3389/fmicb.2019.02916 (PMC6951406; doi:10.3389/fmicb.2019.02916)

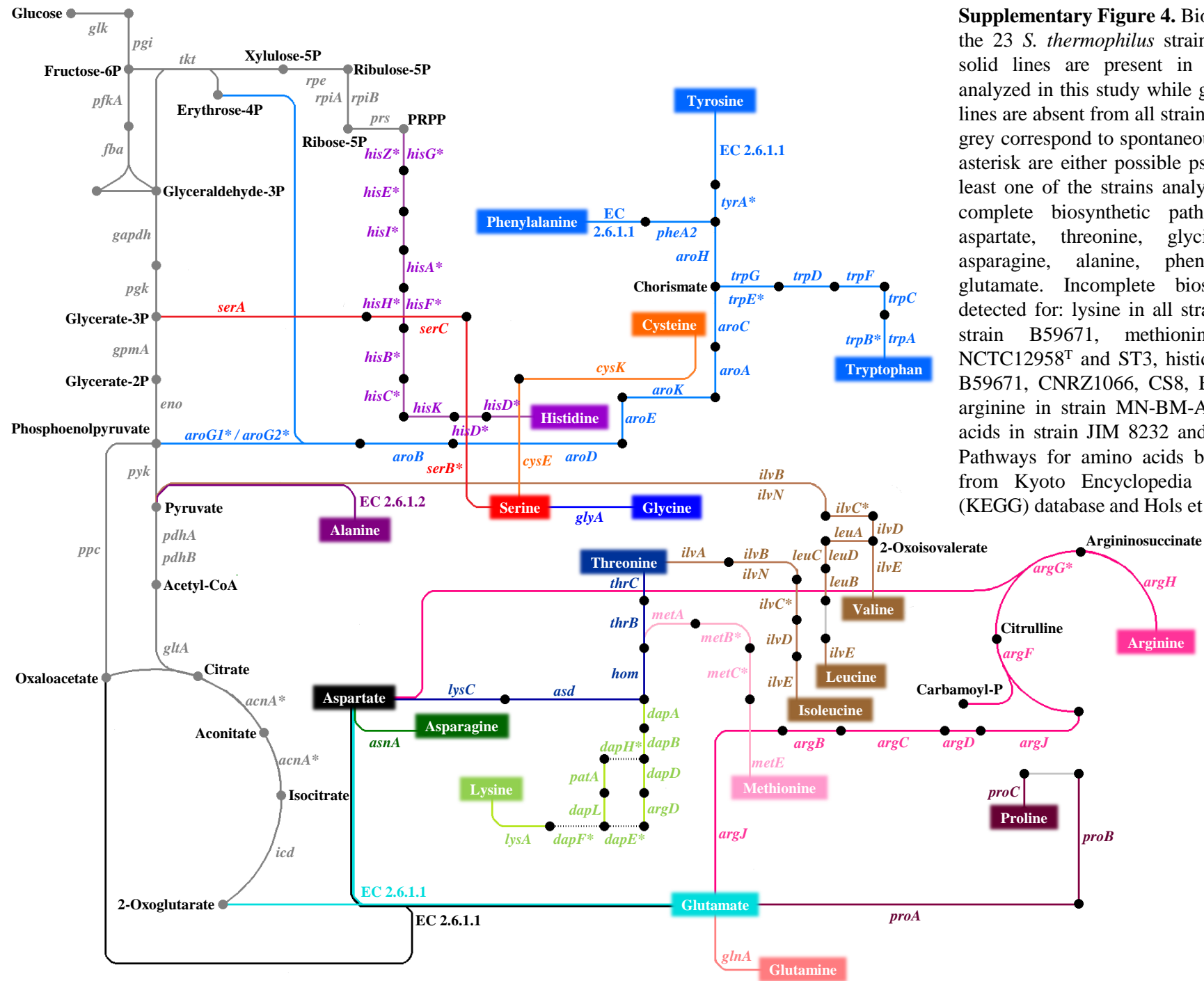

Supplement: Supplementary file 17 [file Data_Sheet_4.PDF]
