# Supplementary material for: Comparative Genomics of Streptococcus thermophilus Support Important Traits Concerning the Evolution, Biology and Technological Properties of the Species
Source: Front Microbiol. 2019 Dec 20;10:2916. doi: 10.3389/fmicb.2019.02916 (PMC6951406; doi:10.3389/fmicb.2019.02916)
Supplement: Supplementary file 18 [file Data_Sheet_5.PDF]

Supplementary Figure 5. Restriction-modification (R-M) systems in the 23 *S. thermophilus* strains as predicted by REBASE database

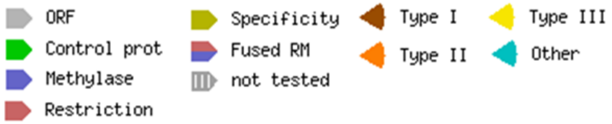

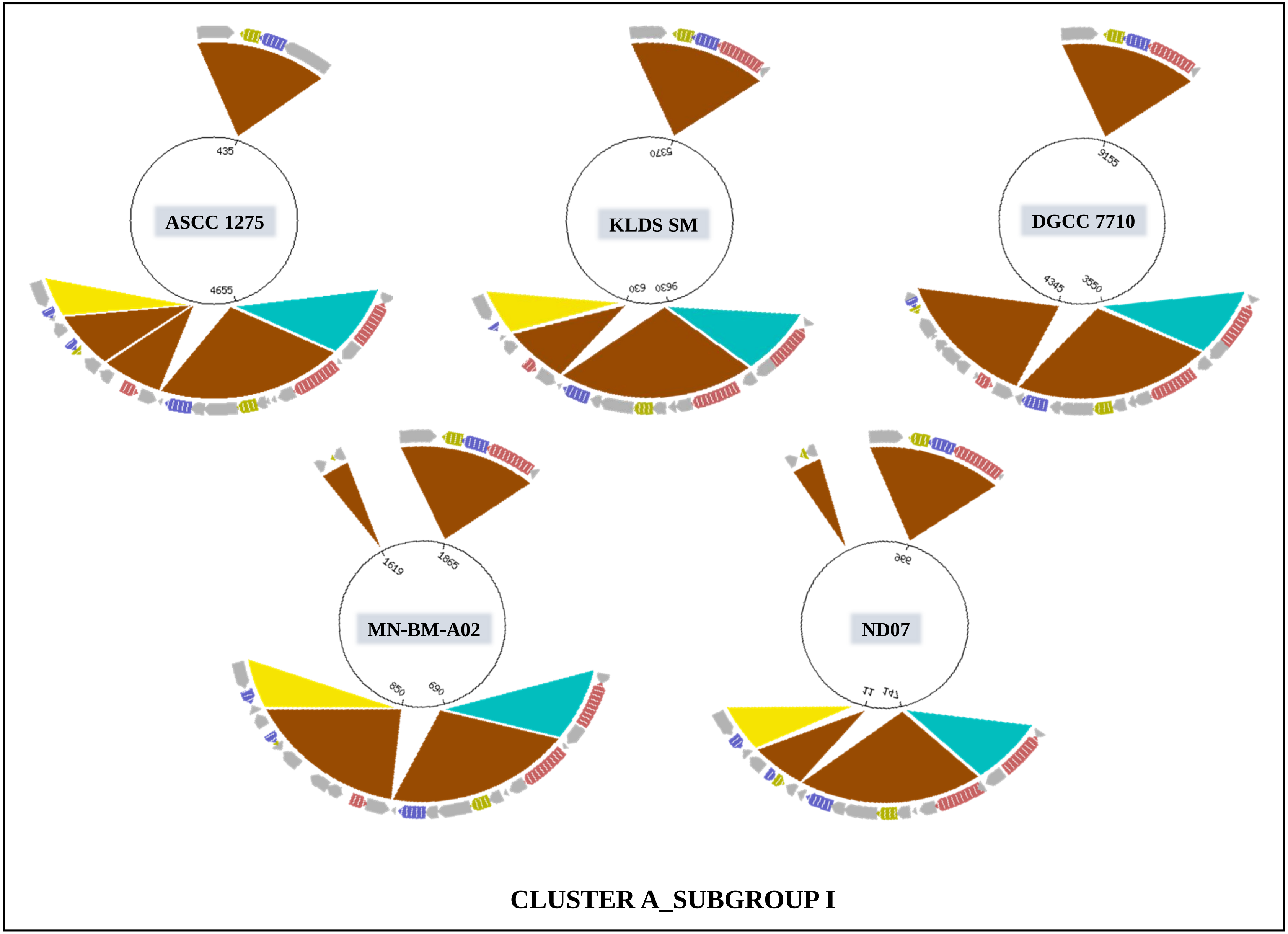

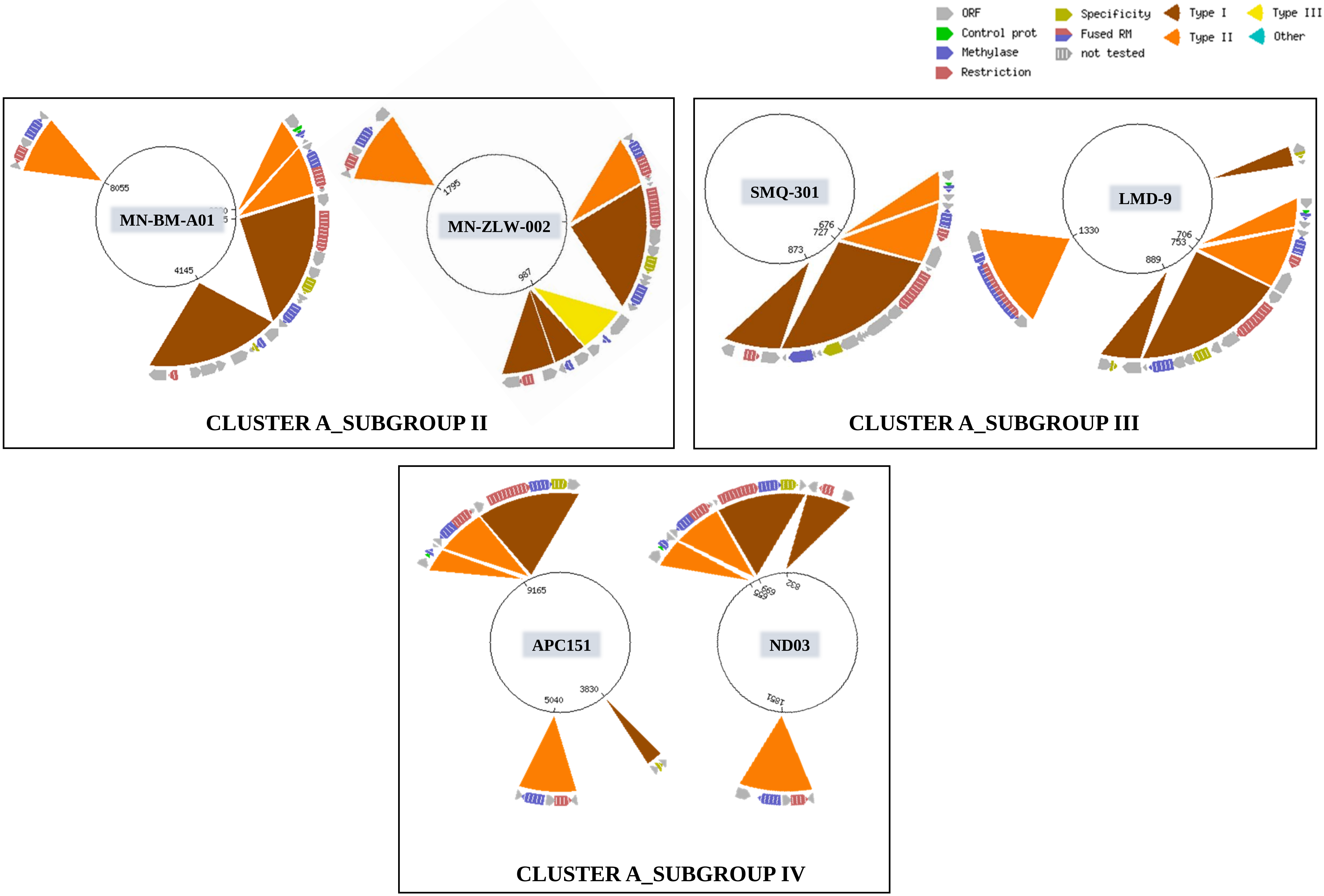
ORF
Control prot
Methylase
Restriction
Specificity
Fused RM
not tested
Type I
Type II
Type III
Other
MN-BM-A01
8055
4145
MN-ZLW-002
1795
987
CLUSTER A_SUBGROUP II
SMQ-301
676
727
873
LMD-9
1330
706
753
889
CLUSTER A_SUBGROUP III
APC151
9165
3830
5040
ND03
699
655
832
1851
CLUSTER A_SUBGROUP IV

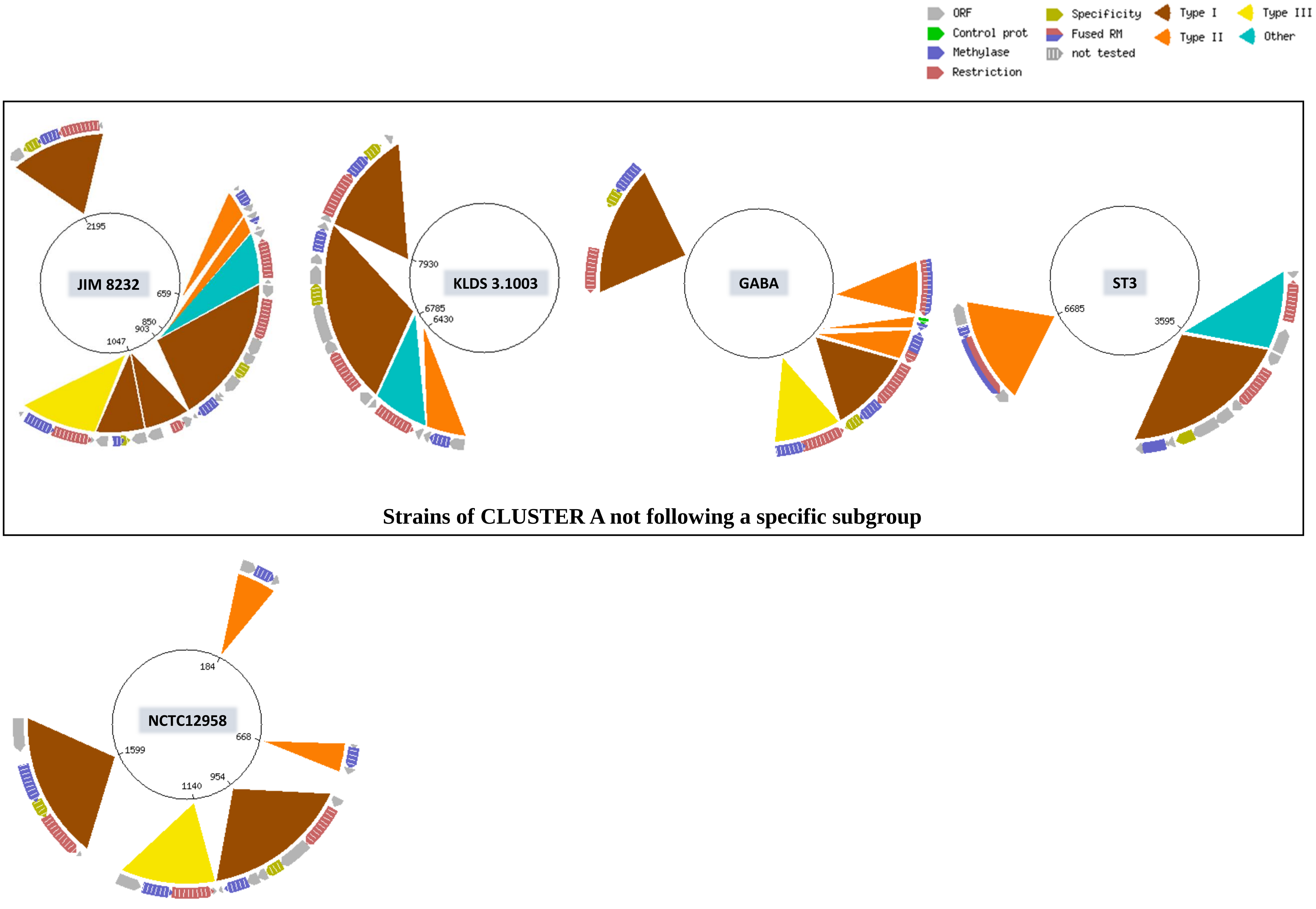
ORF
Control prot
Methylase
Restriction
Specificity
Fused RM
not tested
Type I
Type II
Type III
Other
JIM 8232
2195
659
850
903
1047
KLDS 3.1003
7930
6785
6430
GABA
ST3
6685
3595
Strains of CLUSTER A not following a specific subgroup
NCTC12958
184
668
1599
954
1140

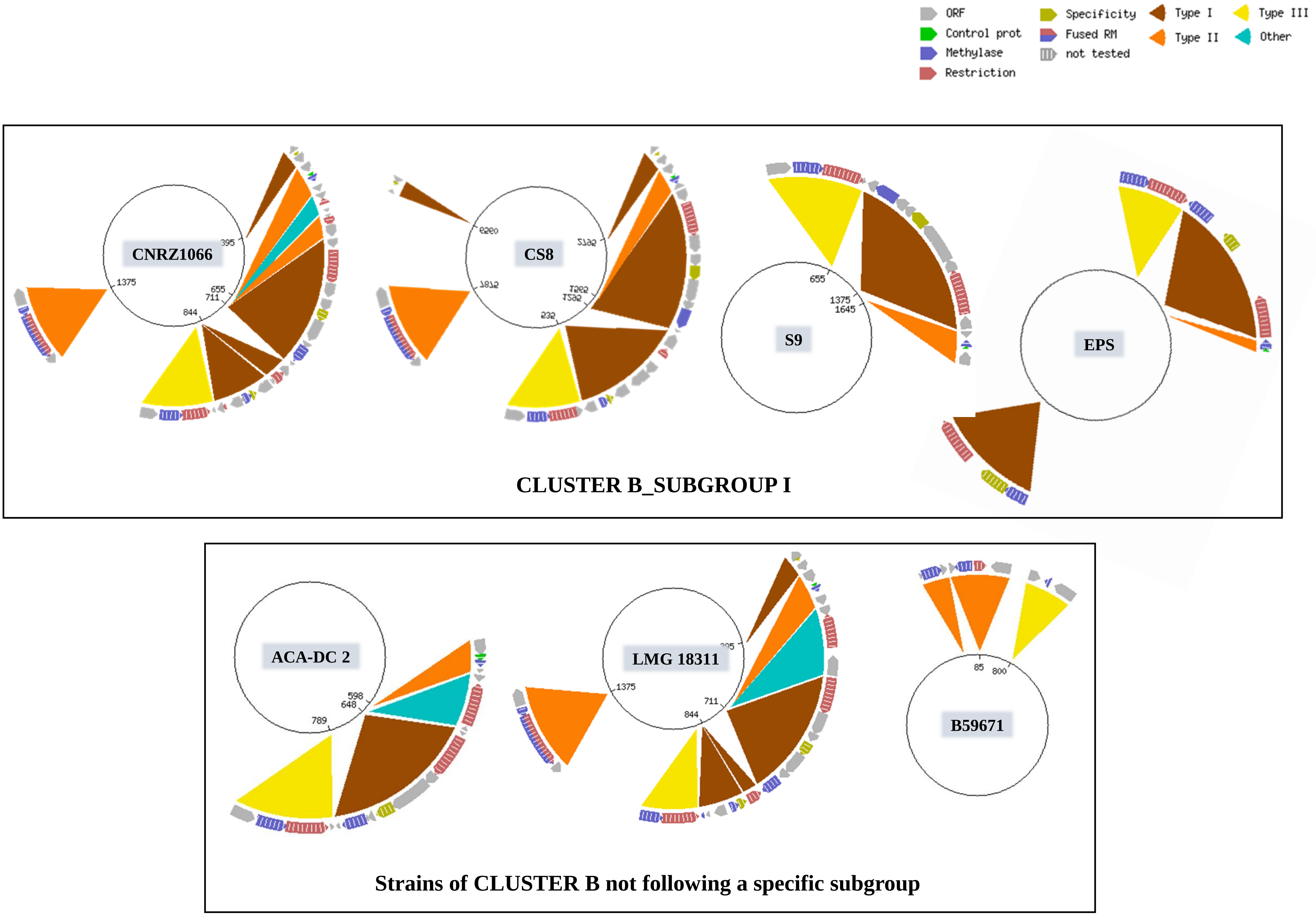
ORF
Control prot
Methylase
Restriction
Specificity
Fused RM
not tested
Type I
Type II
Type III
Other
CNRZ1066
395
1375
655
711
844
CS8
232
S9
655
1375
1645
EPS
CLUSTER B_SUBGROUP I
ACA-DC 2
598
648
789
LMG 18311
1375
711
844
B59671
85
800
Strains of CLUSTER B not following a specific subgroup
