# Supplementary material for: Comparative Genomics of Streptococcus thermophilus Support Important Traits Concerning the Evolution, Biology and Technological Properties of the Species
Source: Front Microbiol. 2019 Dec 20;10:2916. doi: 10.3389/fmicb.2019.02916 (PMC6951406; doi:10.3389/fmicb.2019.02916)
Supplement: Supplementary file 19 [file Data_Sheet_6.pdf]

Supplementary Figure 6. Genomic islands (Gis) in the 23 *S. thermophilus* strains as predicted by IslandViewer 4

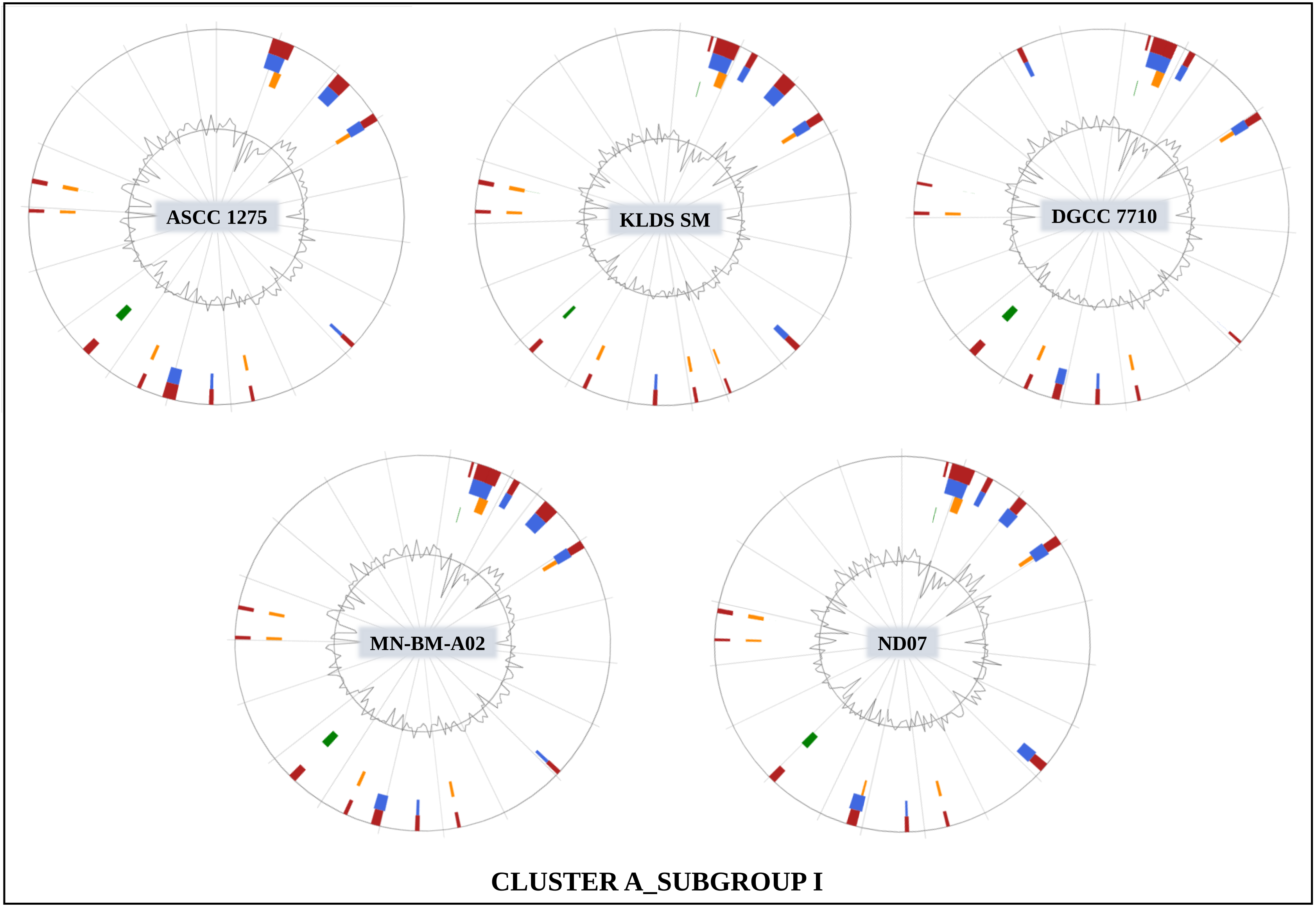

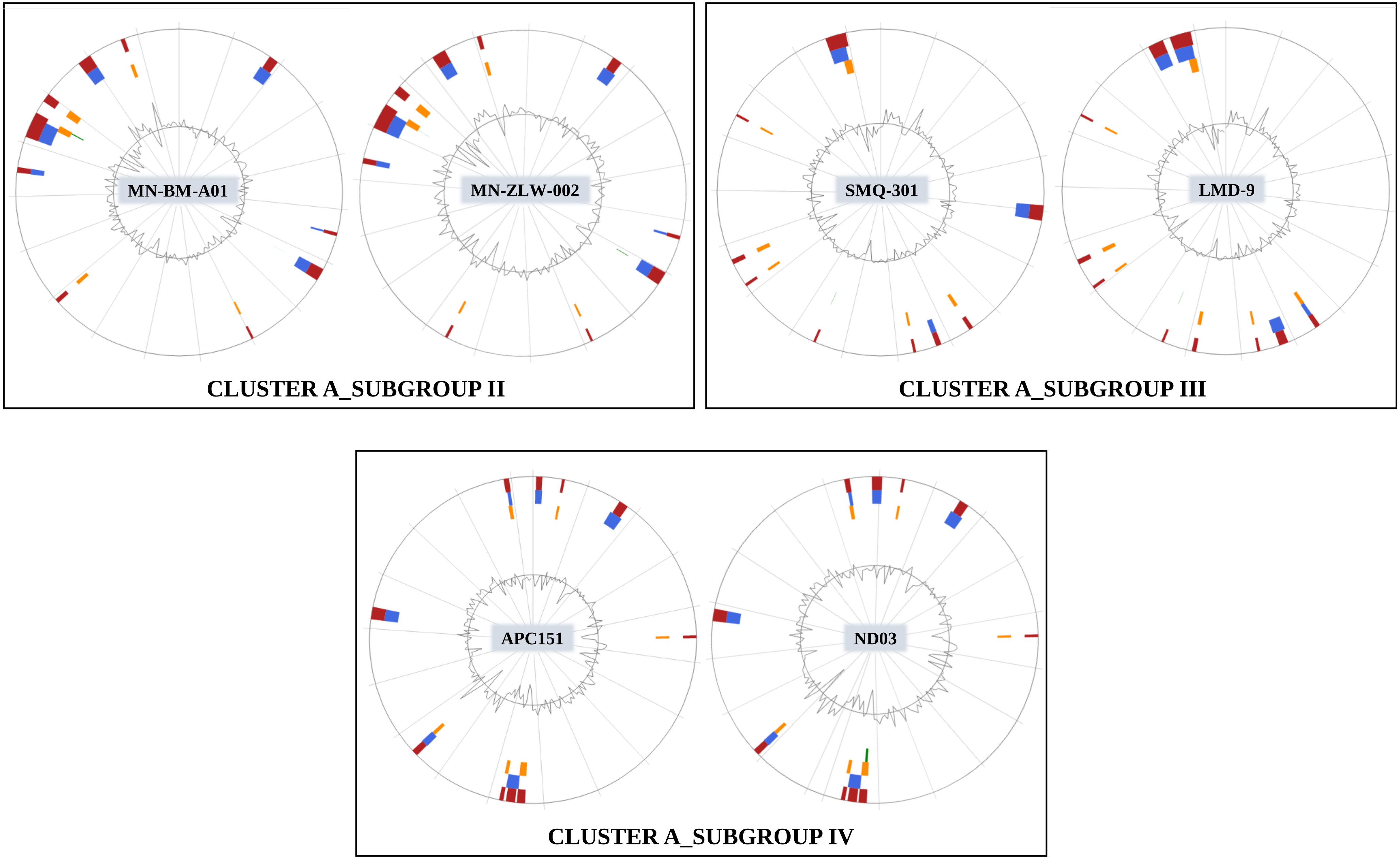
MN-BM-A01
MN-ZLW-002
CLUSTER A_SUBGROUP II
SMQ-301
LMD-9
CLUSTER A_SUBGROUP III
APC151
ND03
CLUSTER A_SUBGROUP IV

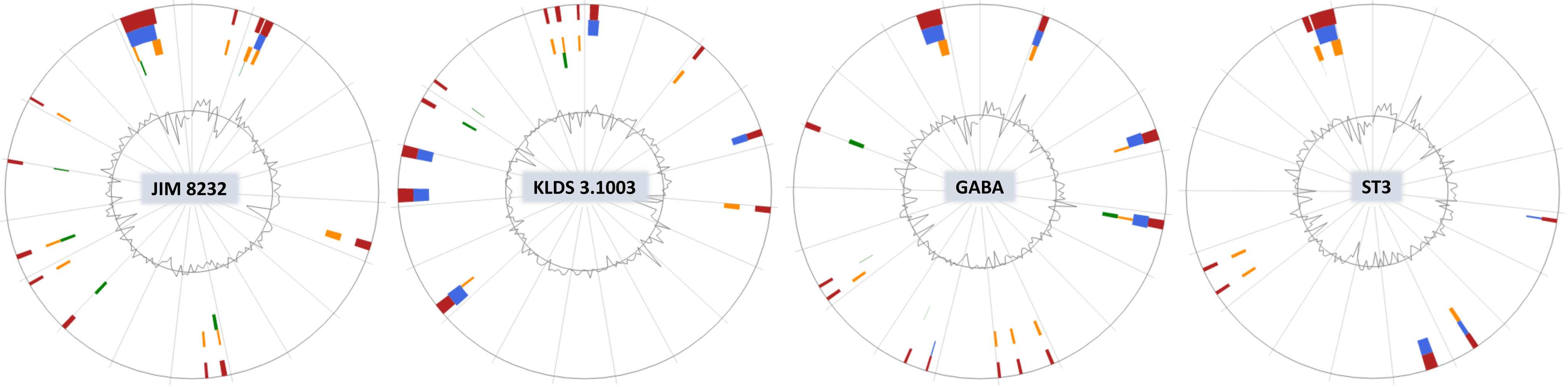

Strains of CLUSTER A not following a specific subgroup

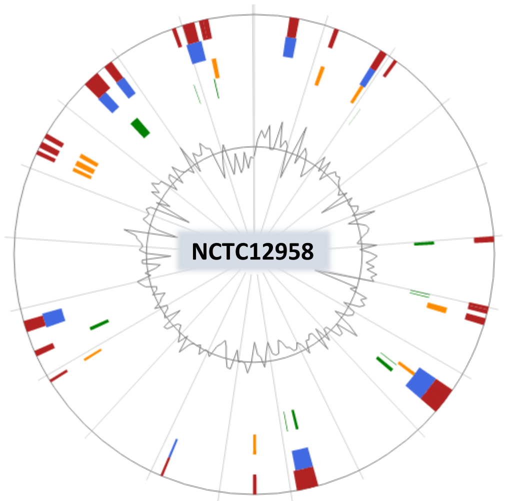

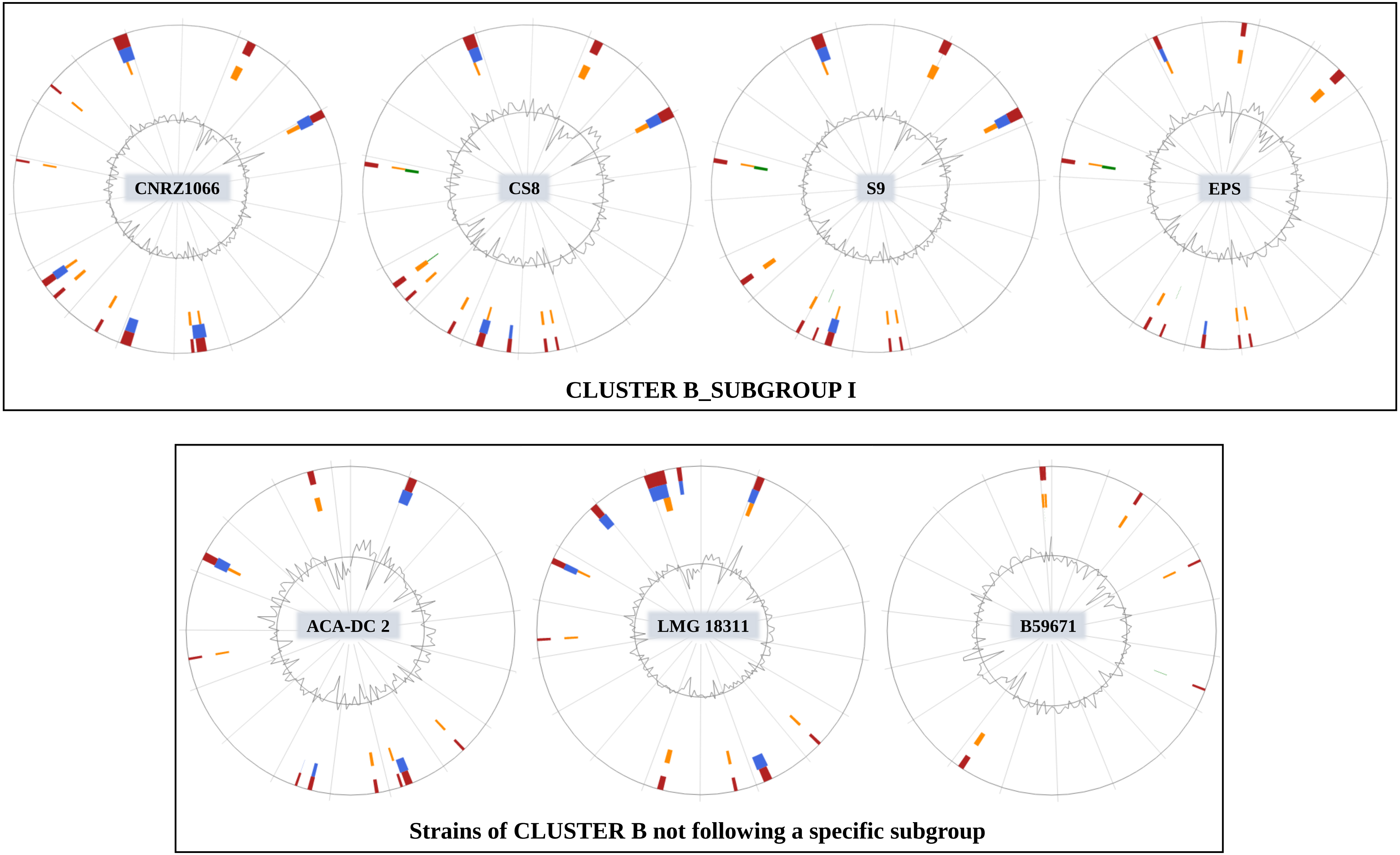
CNRZ1066
CS8
S9
EPS
CLUSTER B_SUBGROUP I
ACA-DC 2
LMG 18311
B59671
Strains of CLUSTER B not following a specific subgroup
